# Al Promotion of In_2_O_3_ for CO_2_ Hydrogenation to Methanol

**DOI:** 10.1021/acscatal.3c04620

**Published:** 2023-11-22

**Authors:** Liang Liu, Brahim Mezari, Nikolay Kosinov, Emiel J. M. Hensen

**Affiliations:** Laboratory of Inorganic Materials and Catalysis, Department of Chemical Engineering and Chemistry, Eindhoven University of Technology, P.O. Box 513, 5600 MB Eindhoven, The Netherlands

**Keywords:** CO_2_ hydrogenation, In_2_O_3_, methanol, doping, aluminum

## Abstract

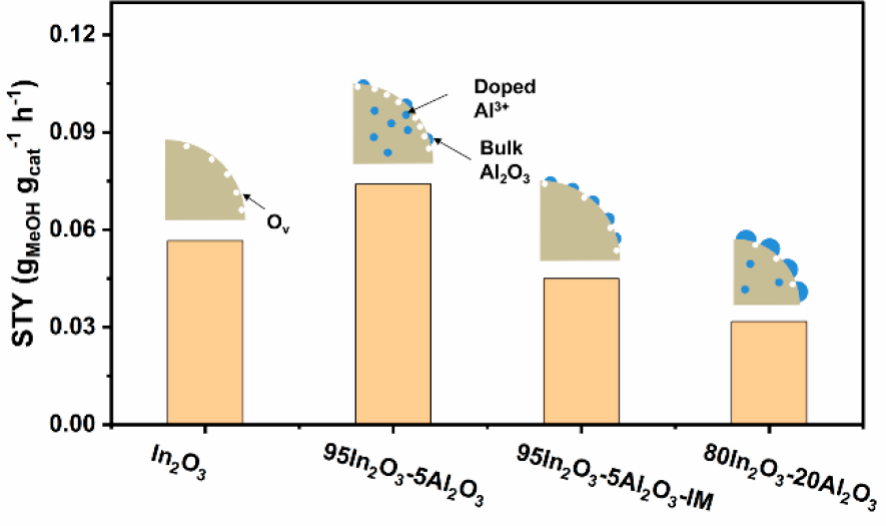

In_2_O_3_ is a promising catalyst for the hydrogenation of CO_2_ to methanol, relevant to renewable energy storage in chemicals. Herein, we investigated the promoting role of Al on In_2_O_3_ using flame spray pyrolysis to prepare a series of In_2_O_3_–Al_2_O_3_ samples in a single step (0–20 mol % Al). Al promoted the methanol yield, with an optimum being observed at an Al content of 5 mol %. Extensive characterization showed that Al can dope into the In_2_O_3_ lattice (maximum ∼ 1.2 mol %), leading to the formation of more oxygen vacancies involved in CO_2_ adsorption and methanol formation. The rest of Al is present as small Al_2_O_3_ domains at the In_2_O_3_ surface, blocking the active sites for CO_2_ hydrogenation and contributing to higher CO selectivity. At higher Al content (≥10 mol % Al), the particle size of In_2_O_3_ decreases due to the stabilizing effect of Al_2_O_3_. Nevertheless, these smaller particles are prone to sintering during CO_2_ hydrogenation since they appear to be more easily reduced. These findings show subtle effects of a structural promoter such as Al on the reducibility and texture of In_2_O_3_ as a CO_2_ hydrogenation catalyst.

## 1 Introduction

Hydrogenation of carbon dioxide (CO_2_) to chemicals and fuels with renewable hydrogen can contribute to mitigating climate change by closing carbon cycles. Methanol is one of the most promising CO_2_ hydrogenation products, as it is both a fuel and a versatile chemical intermediate for producing light olefins, aromatics, and other chemicals.^1^ This explains its central role in the circular methanol economy proposed by Olah.^2^

The Cu/ZnO/Al_2_O_3_ catalyst is widely employed as a commercial catalyst for methanol synthesis from synthesis gas.^3^ Nevertheless, it exhibits relatively low methanol selectivity in the CO_2_ hydrogenation to methanol reaction due to its high activity in the competing reverse water–gas shift (rWGS) reaction.^4^ Moreover, it remains challenging to modify Cu/ZnO/Al_2_O_3_ for the direct hydrogenation of CO_2_ to methanol due to rapid deactivation as a result of active phase sintering.^5,6^ Recently, indium oxide (In_2_O_3_) has emerged as an alternative catalyst for CO_2_ hydrogenation to methanol with reasonable activity and high methanol selectivity.^7^ Promoters, such as Rh, Pt, Pd, Co, Ni, and Au, have already been explored to improve the performance of In_2_O_3_ catalysts.^8−15^ Most of these studies on the use of promoters have focused on enhancing H_2_ activation and CO_2_ adsorption. Li et al. reported that modification of In_2_O_3_ with Rh led to a higher CO_2_ conversion rate and methanol selectivity (methanol yield up to 1 g_MeOH_·g^–1^·h^–1^ at a H_2_/CO_2_ ratio of 3) than a commercial Cu/ZnO/Al_2_O_3_ catalyst.^16^ Doping Pd atoms into the lattice of In_2_O_3_ enhanced H_2_ activation and water desorption, explaining the observed increase in activity and methanol selectivity compared to In_2_O_3_.^14^ Our group reported similar improvements using Ni as a dopant for In_2_O_3_ prepared by flame spray pyrolysis (FSP).^17^ Others have mentioned that promoters can also enhance the ability of the surface to adsorb and activate CO_2_ and stabilize particular reaction intermediates. Shi et al. emphasized the role of interfacial sites between Cu_2_In alloy and In_2_O_3_ in a core–shell CuIn@SiO_2_ catalyst.^18^ The presence of such interfacial sites led to more oxygen vacancies (O_v_), which are sites for CO_2_ adsorption, and a higher methanol yield than In@SiO_2_ catalysts. In general, it is thought that the promoter metals that contribute to H_2_ activation are present in a reduced form in the active catalyst. Although the addition of these promoters can significantly improve the formation of methanol, their exact role has not yet been resolved. This is likely due to the variation in preparation methods employed and the fact that the exact nature of the active sites is not known yet.

Recently, Pérez-Ramírez’s group prepared a series of metal-doped In_2_O_3_ catalysts via FSP to investigate the impact of metal dopants (M = Ag, Au, Co, Ir, Ni, Pd, Pt, Rh, and Ru) on the hydrogenation of CO_2_ to methanol.^19^ It was found that the high dispersion of the metal promoter and proximity to In_2_O_3_ are pivotal to improving methanol synthesis. The authors showed that In can be partially reduced, resulting in alloy phases with the metal dopants. Density functional theory calculations emphasize the ability of these alloys to activate H_2_. Resolving the structure of such In-containing alloys is challenging, because they may vary in size and composition. The presence of small amounts of reduced In in such alloys on In_2_O_3_ hampers the characterization of very small particles by techniques such as EXAFS. Overall, it remains difficult to ascertain the nature of the active sites formed upon metal addition and their exact role in oxygen vacancy formation and CO_2_ conversion.

Next to the promoting role of metals for H_2_ and CO_2_ activation, the methanol yield can also be improved by adding structural promoters, which stabilize the dispersion of the active phase. For instance, ZrO_2_ has been widely used for this purpose as it stabilizes a high In_2_O_3_ dispersion.^7^ In addition, Frei et al. mentioned that the addition of ZrO_2_ can lead to more O_v_ in In_2_O_3_ due to the lattice mismatch between In_2_O_3_ and monoclinic ZrO_2_.^20^ Tsoukalou et al. reported that the formation of In_2_O_3_–ZrO_2_ solid solutions plays an important role in increasing the catalytic activity.^21^ By studying uniformly sized colloidal nanoparticles of In_2_O_3_ on ZrO_2_, the authors investigated the role of different ZrO_2_ phases on the dispersion of In_2_O_3_. When supported on tetragonal or amorphous ZrO_2_, the In_2_O_3_ particles were partially or entirely reduced to metallic In, leading to low activity. In contrast, the formation of a solid solution of In_2_O_3_ with monoclinic ZrO_2_ resulted in very highly dispersed In^3+^ ions, preventing reduction to metallic In and yielding highly active samples. Thus, it was argued that such species can reduce to In^2+^ related to the formation of O_v_, thus explaining the improved activity.

Alumina is the most used support material to stabilize active phases in heterogeneous catalysis.^22−25^ In the context of CO_2_ hydrogenation, it has been found that Al can improve the methanol yield of Pd/ZnO due to enhanced CO_2_ adsorption.^26^ In this work, we investigated the utility of Al as a promoter for In_2_O_3_ in the hydrogenation of CO_2_ to methanol. For this purpose, we deployed FSP to prepare small In_2_O_3_ particles doped with Al. While pure Al_2_O_3_ did not show activity in CO_2_ hydrogenation to methanol, doping small amounts of Al into In_2_O_3_ increased the methanol yield compared to that of pure In_2_O_3_. The morphologies of the FSP-derived materials were characterized by XRD, N_2_ physisorption, and electron microscopy. The coordination environment of Al was investigated using ^27^Al MQMAS NMR. Temperature-programmed desorption of CO_2_ and XPS were used to understand the role of Al doping in improved catalytic performance. Alumina-supported In_2_O_3_ reference samples were prepared by impregnation.

## 2 Experimental Section

### 2.1 Preparation

In_2_O_3_ and Al-doped In_2_O_3_ with different Al content (2 mol %, 5 mol %, 10 mol %, and 20 mol % Al) were synthesized by FSP using a Tethis NPS10 setup. The precursor solution was made by dissolving appropriate amounts of In(NO_3_)_3_·*x*H_2_O (99.999%, Alfa Aesar) and Al(NO_3_)_3_·9H_2_O (99%, Sigma-Aldrich) in a 1:1 (vol %) solvent mixture of ethanol (HPLC, Sigma-Aldrich) and 2-ethylhexanoic acid (99%, Sigma-Aldrich) at room temperature. The total metal concentration (In + Al) was 0.15 mol/L. The precursor solution was injected into the flame at a flow rate of 1.0 mL/min. The flame was sustained with a flow of 1.5 L/min methane and 3.0 L/min oxygen with an additional 5.0 L/min oxygen dispersion flow. Under these conditions, the metal precursors were converted into metal oxides and collected on a glass-fiber filter (Hahnemühle FineArt GmbH, GF6, 257 mm diameter) placed above the FSP nozzle. The catalysts are denoted as *x*In_2_O_3_-*y*Al_2_O_3_, where *x* and *y* represent the molar In and Al content, respectively.

A reference 95In_2_O_3_-5Al_2_O_3_-IM catalyst was prepared by wet impregnation. An aqueous Al(NO_3_)_3_·9H_2_O solution was impregnated on In_2_O_3_ obtained by FSP. The solution was stirred overnight, dried in a rotary evaporator, and calcined at 300 °C for 3 h.

### 2.2 Catalyst Characterization

#### Nitrogen Physisorption

The textural properties of the as-prepared catalysts were analyzed by N_2_ physisorption at −196 °C using a Micrometrics TriStar II 3020 instrument. Before measurement, samples were pretreated overnight at 120 °C under a nitrogen flow. The Brunauer–Emmett–Teller (BET) method was used to determine the specific surface area.

#### Transmission Electron Microscopy (TEM)

The average size and size distribution of the primary particles in the as-prepared and used catalysts were determined by TEM using an FEI Tecnai (type Sphera) instrument operating at an acceleration voltage of 200 kV. For sample preparation, appropriate amounts of samples were dispersed in ethanol under ultrasonic exposure and deposited on holey Cu grids. STEM images and STEM-EDX maps of the used In_2_O_3_–Al_2_O_3_ catalysts were acquired using an aberration-corrected FEI Titan TEM instrument operated at an acceleration voltage of 300 kV.

#### X-ray Diffraction (XRD)

XRD patterns were recorded using a Bruker D2 Phaser diffractometer with Cu Kα radiation (1.5406 Å) between 10 and 90° with a step size of 0.02° at a 0.23 s/step scan rate.

#### Inductively Coupled Plasma Optical Emission Spectroscopy (ICP-OES)

The metal composition of the as-prepared catalysts was analyzed by ICP-OES with a Spectro CIROS CCD Spectrometer. Before measurements, the In_2_O_3_–Al_2_O_3_ catalysts were dissolved in an equivolumetric mixture of nitrate acid and water at room temperature.

#### Temperature-Programmed Reduction (H_2_-TPR)

The reduction of the as-prepared samples was analyzed by H_2_-TPR using a Micromeritics AutoChem II setup equipped with a thermal conductivity detector (TCD). Typically, about 100 mg of the sample was loaded into a quartz U-tube between two quartz wool layers. The sample was pretreated in a He flow (50 mL/min) at 120 °C for 1 h before the measurements. The TPR profile was recorded by heating the sample from 40 to 800 °C at a 10 °C/min rate in a 4 vol % H_2_ in He flow (50 mL/min). H_2_ consumption was monitored with a thermal conductivity detector (TCD). H_2_ consumption was determined by integrating the area under the H_2_-TPR profile in a specific temperature range, and this area was compared to a reference H_2_-TPR experiment in which a known amount of CuO was reduced, assuming the complete reduction of all Cu^2+^ to Cu(0).

#### Temperature-Programmed Desorption CO_2_ (CO_2_-TPD)

The CO_2_ adsorption capacity of the as-prepared samples was determined by CO_2_-TPD using a plug flow reactor setup coupled with a mass spectrometer (Balzers TPG251). Before the CO_2_-TPD analysis, the samples were pretreated in a reaction mixture (CO_2_/H_2_/N_2_ = 10:30:10 mL/min, 30 bar) at 260 °C for 12 h, followed by a switch to a mixture of H_2_ and N_2_ (H_2_/N_2_ = 30:20 mL/min) at 260 °C. Afterward, the sample was sealed with two three-way valves and transferred to a glovebox. It was loaded into a quartz reactor without air exposure for the CO_2_-TPD experiments. The CO_2_ adsorption was conducted in pure CO_2_ (30 mL/min) at 50 °C for 2 h. Then, the reactor was purged with He for 1 h to remove the weakly adsorbed CO_2_. The TPD step involved heating in a He flow of 30 mL/min from 50 to 600 °C at a 10 °C/min rate. To distinguish between surface carbonates and CO_2_ adsorbed on oxygen vacancies, a representative sample (95In_2_O_3_–Al_2_O_3_) was pretreated in two ways, namely with and without O_2_ treatment at 260 °C for 1 h before CO_2_ adsorption. The TPD procedure was performed in the same way as that outlined above. The desorbed CO_2_ was quantitatively analyzed by integrating the area under the desorption profile. To determine the absolute amount of CO_2_ desorbed from the CO_2_ TPD profile, we calibrated the CO_2_ signal by decomposing known amounts of MgCO_3_ in a TPD experiment. From this, a calibration line relating the amount of desorbed CO_2_ to the peak area was constructed, which was used to determine the amount of desorbed CO_2_ in the other experiments.

#### X-ray Photoelectron Spectroscopy (XPS)

The surface chemical properties of the as-prepared and used catalysts after CO_2_ hydrogenation were studied using a K-Alpha XPS instrument (Thermo Scientific) with a monochromatic X-ray source and a 180° double focusing hemispherical analyzer. The samples were placed onto a double-sided carbon tape in a glovebox and transferred to the spectrometer via a gastight transfer holder. For XPS analysis of the used catalysts, the catalysts were first operated in the reaction mixture (CO_2_/H_2_/N_2_ = 10:30:10 mL/min) at 260 °C and 30 bar for 14 h. The reactor was then depressurized at 260 °C, cooled to room temperature in a 50 mL/min N_2_ flow, sealed with two three-way valves, and transferred to a glovebox for sample storage and preparation for XPS analysis without air exposure. Spectra were collected using an aluminum anode (Al Kα = 1486.68 eV) operating at 72 W and a spot size of 400 μm. Survey scans were measured at a constant pass energy of 200 eV, and region scans were measured at 50 eV. The spectra were analyzed using the CasaXPS software (version 3.2.23), and energy calibration was performed against the C 1s peak of adventitious carbon at a binding energy of 284.8 eV.

#### Solid-State Nuclear Magnetic Resonance (NMR) Spectroscopy

NMR measurements were performed on a 11.7 T Bruker AvanceNeo 500 spectrometer operating at 132 MHz for ^27^Al. The NMR measurements were carried out using a 2.5 mm solid-state MAS probe head with a sample rotation rate of 25 kHz. ^27^Al NMR spectra were recorded with a single-pulse sequence with an 18° pulse duration of 1 μs and an interscan delay of 1 s. MQMAS spectra were recorded by a three-pulse sequence p_1_–t_1_–p_2_–τ–p_3_–t_2_ for triple-quantum generation and zero-quantum filtering (strong pulses p_1_ = 3.4 and p_2_ = 1.4 μs at a nutation frequency ν_1_ = 100 kHz, a soft pulse p_3_ = 11 μs at ν_1_ = 8 kHz, a filter time τ = 20 μs, and an interscan delay of 0.5 s). The NMR chemical shift of ^27^Al was done using a saturated Al(NO_3_)_3_ solution.

#### Infrared Spectroscopy (IR)

Infrared spectra were collected by using a Nicolet FT-IR spectrometer equipped with a cryogenic MCT detector. The IR experiments were performed in an in situ homemade cell equipped with CaF_2_ windows. The samples were initially diluted with FSP-made ZrO_2_ at a weight ratio of 1/10, then pressed into pellets, and pretreated in He at 260 °C for 1 h. The background was collected after cooling to 50 °C in He. The in situ high-pressure experiments were performed at 10 bar in a CO_2_/H_2_ mixture (1:3, total flow 50 mL/min). The reaction temperature was heated from 50 to 260 °C. Once a steady state was reached at 260 °C, the reaction was maintained for 1 h. The spectra were collected throughout the temperature-programmed and reaction processes at 260 °C. To track the changes in intermediates adsorbed on In_2_O_3_, the gas was switched from the reactant gas mixture (CO_2_:H_2_ = 1:3, 50 mL/min, 10 bar) to pure H_2_ (50 mL/min, 1 bar) at 260 °C, and time-resolved IR spectra were collected for 1 h. For the temperature-programmed desorption (TPD) procedure in He, the system was cooled to 50 °C after temperature-programmed CO_2_ hydrogenation. Then, the gas was switched from the reactant gas mixture (CO_2_:H_2_ = 1:3, 50 mL/min, 10 bar) to He (100 mL/min, 1 bar) at 50 °C, and IR spectra were collected throughout the temperature-programmed process from 50 to 400 °C in He.

### 2.3 Catalytic Activity Measurements

The catalytic performance of the catalysts in CO_2_ hydrogenation was evaluated at 260 °C and 30 bar using a downflow stainless-steel reactor (ID = 4 mm). Typically, the catalyst was sieved into 125–250 μm, loaded into the reactor (100 mg), and pretreated at 260 °C at a rate of 5 °C/min at 1 bar for 1 h in a He flow of 50 mL/min. After pretreatment, the catalyst was exposed to a reaction mixture (CO_2_/H_2_/N_2_ = 10:30:10 mL/min), and the pressure in the reactor was increased to 30 bar using a back-pressure regulator. The outlet gas line was kept at 130 °C to prevent condensation of the reaction products. The product composition was analyzed by online gas chromatography (Interscience, CompactGC) equipped with Rtx-1 (FID), Rt-QBond and Molsieve 5A (TCD), and Rt-QBond (TCD) columns. Catalytic measurements were typically performed for ca. 12 h, during which a steady state was reached. CO_2_ conversion (*X*), product selectivity (*S*), and product formation rate (*r*) were calculated using the following equations
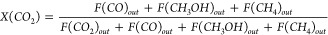
1
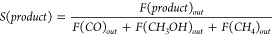
2
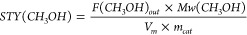
3where *F* stands for the volumetric flow rate determined based on the N_2_ internal standard using calibrated response factors, *Mw*(*CH_3_OH*) is the molecular weight of methanol, and *V_m_* is the molar volume of an ideal gas at standard conditions.

## 3 Results and Discussion

### 3.1 Characterization of As-Prepared In_2_O_3_-Al_2_O_3_

**Figure 1:**
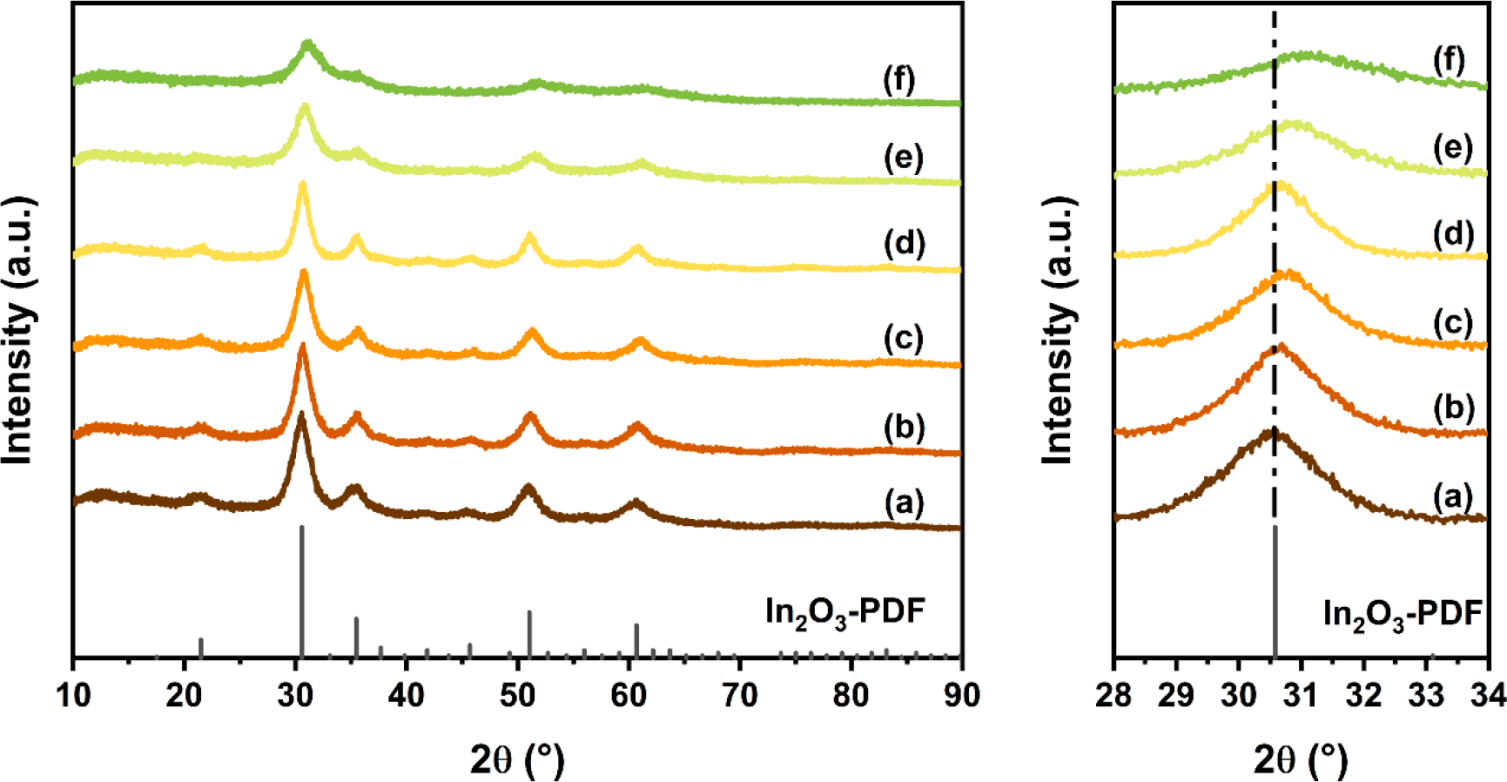
XRD patterns of the as-prepared (a) In_2_O_3_, (b) 98In_2_O_3_-2Al_2_O_3_, (c) 95In_2_O_3_-5Al_2_O_3_, (d) 95In_2_O_3_-5Al_2_O_3_-IM, (e) 90In_2_O_3_-10Al_2_O_3_, and (f) 80In_2_O_3_-20Al_2_O_3_ samples.

A series of Al-doped In_2_O_3_ and In_2_O_3_ catalysts were prepared by FSP. XRD patterns of the as-prepared samples are collected in Figure 1. The diffraction lines of all samples can be related to the cubic structure of In_2_O_3_. No features of other crystalline phases were observed, pointing to a high dispersion of Al in In_2_O_3_ in the Al-containing samples. The slight shift to higher angles of the diffraction lines underpins the substitution of In^3+^ by smaller Al^3+^ ions in the lattice of In_2_O_3_. Above an Al content of 10 mol %, the diffraction lines of In_2_O_3_ substantially broaden, implying a decrease in the In_2_O_3_ particle size. A reference sample was prepared by wet impregnation; i.e., FSP-made In_2_O_3_ was impregnated with an Al(NO_3_)_3_ solution to achieve the same 5 mol % Al loading as the 95In_2_O_3_-5Al_2_O_3_ sample prepared by one-step FSP. The XRD pattern of the resulting 95In_2_O_3_-5Al_2_O_3_-IM sample after calcination is comparable to the XRD patterns of In_2_O_3_ and 95In_2_O_3_-5Al_2_O_3_, indicative of the high dispersion of Al on the surface of In_2_O_3_.

**Figure 2:**
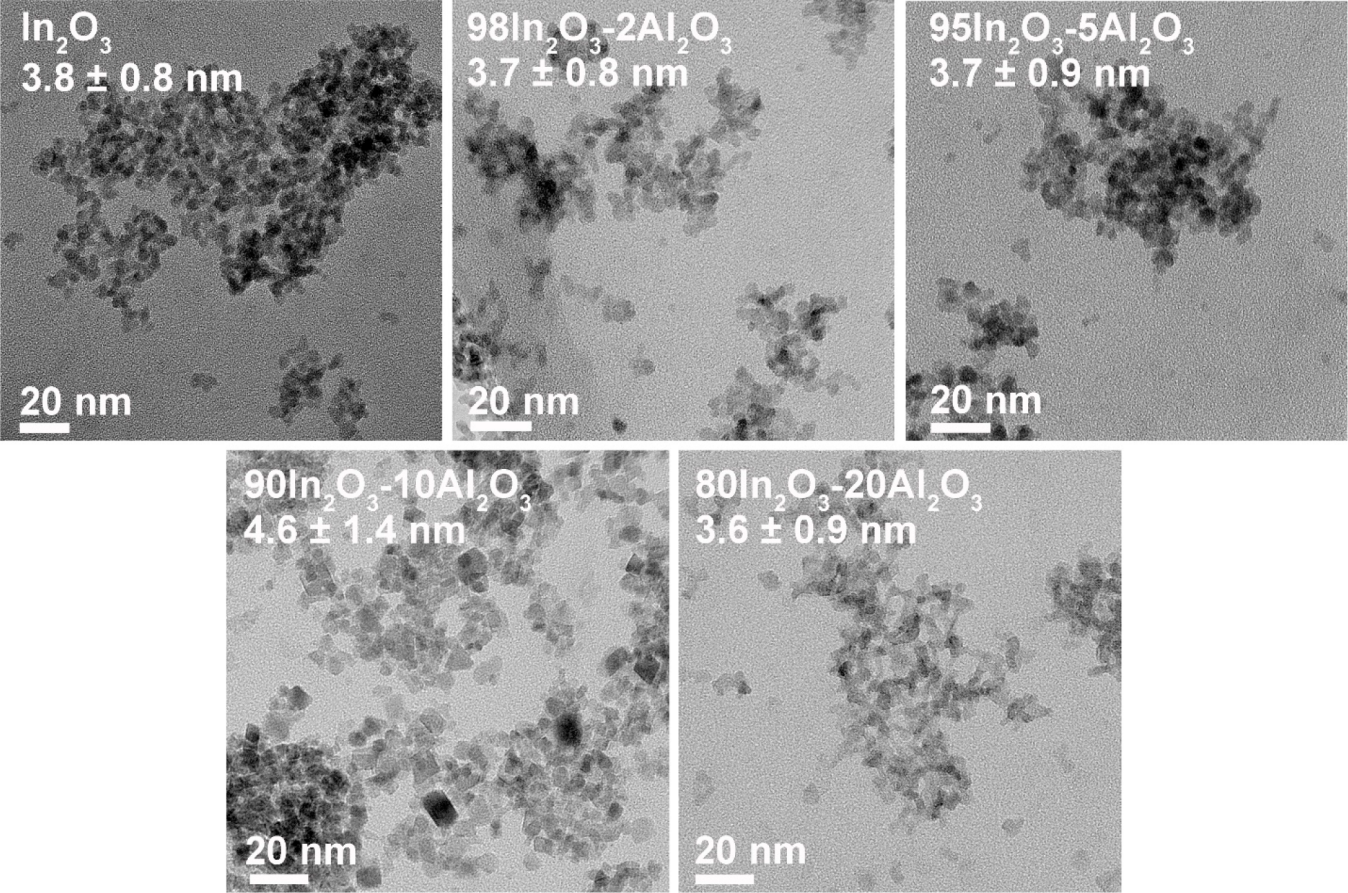
TEM images of the as-prepared In_2_O_3_–Al_2_O_3_ samples.

The particle size and specific surface areas of the as-prepared In_2_O_3_–Al_2_O_3_ samples are summarized in Table 1. The particle size of samples with a low Al content (<5 mol % Al) is approximately 5.5 nm. The In_2_O_3_ particles became slightly smaller with increasing the Al content. For instance, the average particle size of 80In_2_O_3_-20Al_2_O_3_ is 4.2 nm. TEM was used to determine the morphology and particle size of the In_2_O_3_–Al_2_O_3_ samples. All as-prepared In_2_O_3_–Al_2_O_3_ samples show a similar morphology consisting of uniformly sized globular nanoparticles despite the significant differences in chemical composition. The samples are composed of particles in the 3.5–6 nm range (Figure 2). The specific surface area of the FSP-prepared samples ranged from 146 to 171 m^2^·g^–1^. In line with the trend in particle size, the surface area increases with the Al content. The specific surface area of the impregnated sample is only half of that of the 95In_2_O_3_-5Al_2_O_3_ sample. We speculate that the Al_2_O_3_ phase formed upon impregnation and calcination fills some of the interparticle voids, strongly reducing the surface area of the parent In_2_O_3_ support.

**Table 1:** Particle Size and Specific Surface Area of the as-Prepared and Used In_2_O_3_–Al_2_O_3_ Catalysts

	As-prepared^b^		Used^c^	
Sample	d_XRD_ (nm)^a^	Specific surface area (m^2^·g^–1^)	d_XRD_ (nm)^a^	Specific surface area (m^2^·g^–1^)
In_2_O_3_	5.5	150	9.8	76
98In_2_O_3_-2Al_2_O_3_	5.5	153	9.4	69
95In_2_O_3_-5Al_2_O_3_	5.3	146	9.2	73
90In_2_O_3_-10Al_2_O_3_	4.5	162	9.9	72
80In_2_O_3_-20Al_2_O_3_	4.2	171	14.6	83
95In_2_O_3_-5Al_2_O_3_-IM	6.3	83	7.8	60

a Particle size based on XRD line broadening.

b As-prepared samples prepared by FSP.

c After CO_2_ hydrogenation (260 °C, 30 bar, CO_2_/H_2_/N_2_ = 10/30/10 mL/min, 14 h).

**Figure 3:**
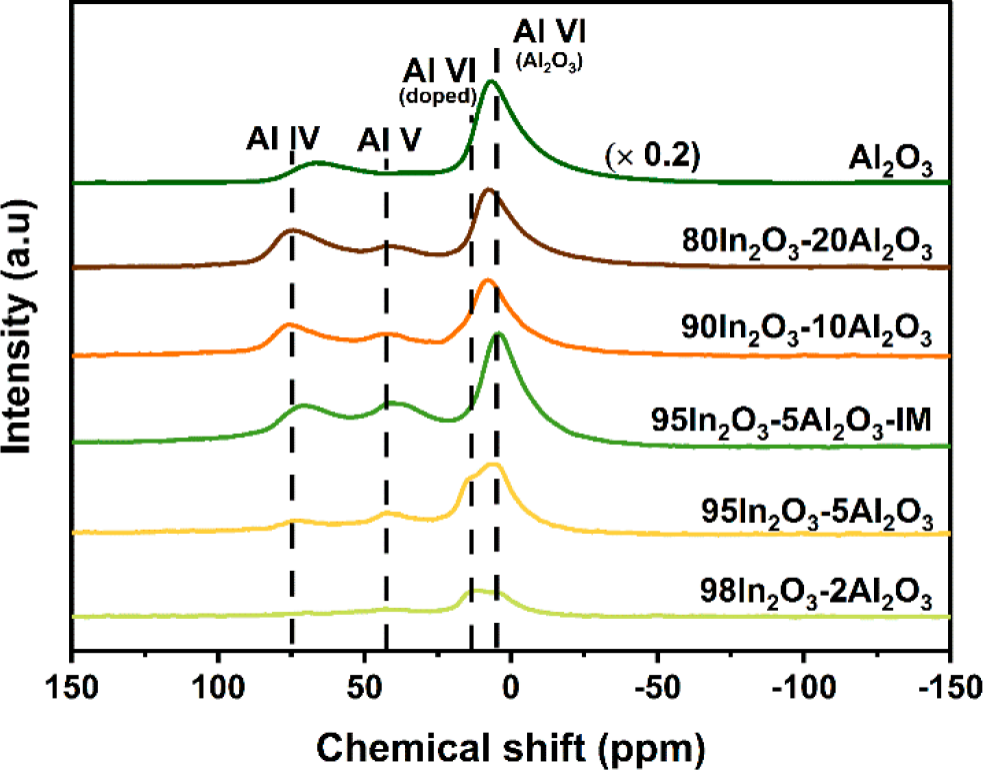
^27^Al NMR of as-prepared In_2_O_3_–Al_2_O_3_ samples with different Al content and Al_2_O_3_.

**Figure 4:**
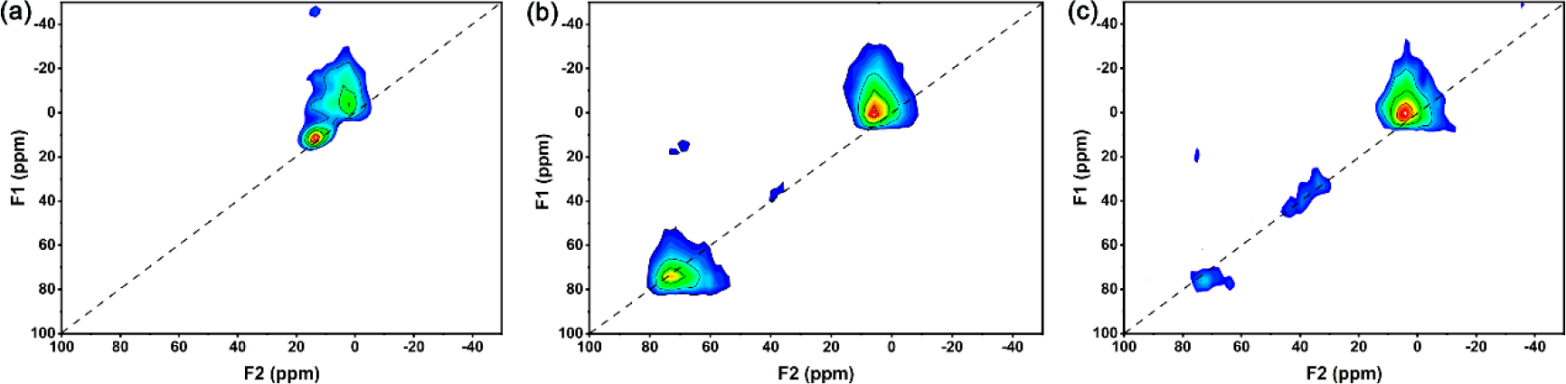
^27^Al MQMAS NMR patterns of the as-prepared (a) 95In_2_O_3_-5Al_2_O_3_, (b) 80In_2_O_3_-20Al_2_O_3_, and (c) 95In_2_O_3_-5Al_2_O_3_-IM samples.

The local environment of the Al^3+^ sites in the various Al-containing samples was investigated by ^27^Al NMR spectroscopy. As shown in Figure 3, the spectra of these samples contain features at 60–75 ppm due to tetrahedral Al (Al^IV^), 35–50 ppm due to distorted tetrahedral or pentacoordinated Al (Al^V^), and 4–14 ppm due to octahedral (Al^VI^).^27,28^ The ^27^Al NMR spectrum of the Al_2_O_3_ sample prepared by FSP (solution of Al(NO_3_)_3_, injection rate 1.0 mL/min) is characterized by Al^IV^ and Al^VI^ features at 5.5 and 65.0 ppm, respectively. XRD shows that γ-Al_2_O_3_ is the dominant phase in FSP-derived Al_2_O_3_ (Figure S1). Accordingly, we attribute the feature in the range of 4.0 to 5.5 ppm observed in the In_2_O_3_–Al_2_O_3_ samples to small Al_2_O_3_ particles that are likely highly dispersed on the In_2_O_3_ surface. In the body-centered cubic bixbyite crystal structure of In_2_O_3_, octahedral sites are occupied by In atoms, while O atoms occupy the tetrahedral sites.^29^ Compared with Al_2_O_3_, the samples with a low Al content contain an additional octahedral Al peak at ∼12 ppm, which we accordingly assign to Al doped into the In_2_O_3_ lattice. This assignment was also put forward by Dogan et al.^30^ The possible presence of pentacoordinated Al (Al^V^) can be understood in terms of the very small Al_2_O_3_ domains interacting with the In_2_O_3_ in analogy with amorphous silica–alumina (ASA). In ASA obtained by homogeneous deposition-precipitation of Al on SiO_2_ followed by calcination, Al^V^ species have been assigned to the interface between Al_2_O_3_ domains and the mixed silica–alumina support.^31^ A strong correlation between Al^V^ probed by ^27^Al NMR spectroscopy and the amount of alumina domains has been considered as support for the relatively small size of such domains in ASA.^32^ To distinguish Al^V^ species from Al^IV^ shifted by a strong quadrupolar distortion in the In_2_O_3_–Al_2_O_3_ samples, we employed ^27^Al MQMAS NMR spectroscopy, which allows distinguishing the chemical-shift heterogeneity from quadrupolar induced peak broadening.^33^ The resulting spectra in Figure 4 show that there are two types of octahedral sites in 95In_2_O_3_-5Al_2_O_3_ samples, confirming the presence of Al_2_O_3_ next to the doping of Al^3+^ in In_2_O_3_ at low Al content. The presence of pentacoordinated Al was also confirmed for the 95In_2_O_3_-5Al_2_O_3_-IM and 80In_2_O_3_-20Al_2_O_3_ samples.

The results of the deconvolution of the 1D ^27^Al NMR spectra are collected in Table 2. As the Al content increases in the FSP-derived samples, the fraction of doped Al^VI^ decreases from 25% to 4%. The absolute amount of doped Al increases from 0.4 to 1.2 mol % Al when the nominal Al content increases from 1.5 to 4.6 mol %. At a higher Al content, the amount of doped Al slightly decreases to a value of 0.8 mol % for 80In_2_O_3_-20Al_2_O_3_. The nearly linear increase in interface Al^V^ species with Al content (Figure S2) points to a high dispersion of the Al_2_O_3_ domains on the In_2_O_3_ support. The trends in the relative and absolute amounts of the different Al species indicate that a small fraction of Al (∼1 mol %) can be dissolved in In_2_O_3_, while most of the Al ends up as small Al_2_O_3_ aggregates on the In_2_O_3_ surface. According to the NMR spectrum of 95In_2_O_3_-5Al_2_O_3_-IM, the sample obtained by impregnation does not contain doped Al^VI^ species. Moreover, the amount of Al^V^ (1.3 mol %) was somewhat higher than that of the corresponding FSP-prepared sample (0.9 mol %), suggesting that impregnation also leads to a highly dispersed Al_2_O_3_ phase on the surface of the In_2_O_3_ support.

**Table 2:** Peak Position and Peak Integral Calculated Using Deconvolution of the 1D ^27^Al NMR Spectra for the In_2_O_3_–Al_2_O_3_ Samples

	Peak position				Peak integral			
			Al^VI^ (ppm)				Al^VI^ (%)	
Sample	Al^IV^ (ppm)	Al^V^ (ppm)	Doped Al	Al_2_O_3_	Al^IV^ (%)	Al^V^ (%)	Doped Al	Al_2_O_3_
Al_2_O_3_	65.0	37.4	-	5.5	20	6	-	74
98In_2_O_3_-2Al_2_O_3_ (fresh)	69.4	40.3	12.0	5.2	9	23	25	43
98In_2_O_3_-2Al_2_O_3_ (used)^a^	66.8	41.2	12.0	5.0	12	23	30	35
95In_2_O_3_-5Al_2_O_3_ (fresh)	71.1	40.4	11.6	5.2	10	20	25	45
95In_2_O_3_-5Al_2_O_3_ (used)^a^	70.3	40.6	12.0	5.2	13	24	30	33
90In_2_O_3_-10Al_2_O_3_ (fresh)	74.1	43.5	11.5	5.2	22	23	10	45
90In_2_O_3_-10Al_2_O_3_ (used)^a^	70.9	38.1	12.4	5.0	8	44	6	42
80In_2_O_3_-20Al_2_O_3_ (fresh)	72.8	42.8	11.5	5.2	31	18	4	47
80In_2_O_3_-20Al_2_O_3_ (used)^a^	71.3	40.6	-	6.6	24	27	-	49
95In_2_O_3_-5Al_2_O_3_-IM (fresh)	69.5	38.9	-	4.0	22	26	-	52
95In_2_O_3_-5Al_2_O_3_-IM (used)^a^	72.2	42.8	-	4.0	32	40	-	28

a After CO_2_ hydrogenation (260 °C, 30 bar, CO_2_/H_2_/N_2_ = 10/30/10 mL/min, 14 h).

### 3.2 Surface Characterization

**Figure 5:**
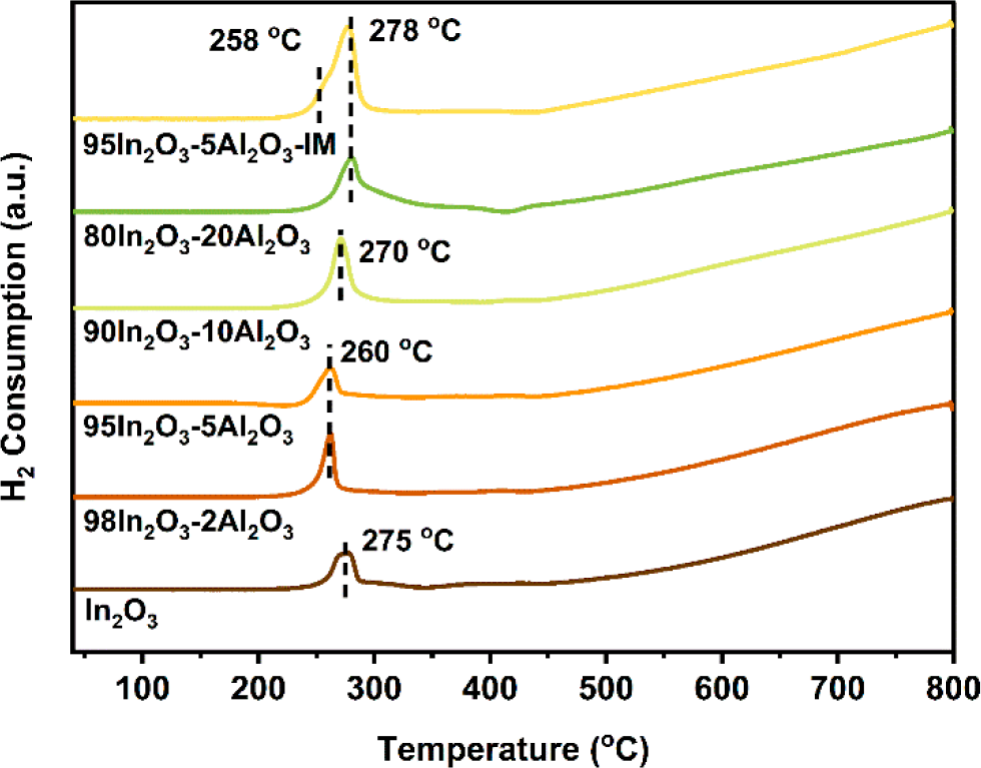
H_2_-TPR profiles of the In_2_O_3_–Al_2_O_3_ samples.

H_2_-TPR was carried out to determine the reducibility of the as-prepared samples (Figure 5). The peak at 275 °C for pure In_2_O_3_ is due to the reduction of the surface of In_2_O_3_, which generates O_v_.^7^ The broad and rising H_2_ uptake above 450 °C is due to the reduction of the bulk of the In_2_O_3_ particles.^7^ Introducing small amounts of Al leads to a shift of the first reduction peak toward lower temperature. In line with an earlier conclusion by Frei et al.,^14^ we explain the more facile reduction to local distortion of the In_2_O_3_ lattice due to doped Al. The position of the first reduction peak shifts to higher temperatures and broadens for Al contents of 10 mol % and higher. We attribute this to predominant Al doping in In_2_O_3_ at low Al content and clustering of Al into small Al_2_O_3_ aggregates at higher Al content. As shown above, the latter aggregates cover part of the In_2_O_3_ surface and stabilize the smaller In_2_O_3_ particles. The latter aspect can explain the more heterogeneous surface reduction observed by H_2_-TPR. The surface reduction feature of the impregnated sample occurs at a higher temperature than that of In_2_O_3_ and at a similar temperature as the reduction feature for 80In_2_O_3_-20Al_2_O_3_. A weak shoulder at a lower temperature of 258 °C in the impregnated sample may be due to a small amount of Al incorporated into In_2_O_3_ during the calcination step.

The H_2_ consumption of the first reduction feature from 200 to 350 °C was normalized to the catalyst weight. The normalized H_2_ consumption increases with the Al content (Table S1). For instance, the H_2_ consumption normalized by catalyst weight increases from 0.35 mmol/g_cat_ for In_2_O_3_ to 0.45 and 0.65 mmol/g_cat_ for 95In_2_O_3_-5Al_2_O_3_ and 80In_2_O_3_-20Al_2_O_3_, respectively. The H_2_ consumption for the 95In_2_O_3_-5Al_2_O_3_-IM sample was even higher at 0.90 mmol/g_cat_. In addition, we deconvoluted this TPR peak for the impregnated sample to calculate the H_2_ consumption corresponding to the shoulder peak area at ∼258 °C (Figure S3). The corresponding H_2_ consumption is ∼0.19 mmol/g_cat_, which is approximately half of the H_2_ consumption for the FSP-derived 95In_2_O_3_-5Al_2_O_3_ sample (Table S1). Given that the H_2_ consumption is determined by integrating the area of the reduction peak caused by doped Al, we can estimate the absolute amount of doped Al in the 95In_2_O_3_-5Al_2_O_3_-IM sample, which is about half of the 1.2 mol % determined for the FSP-derived 95In_2_O_3_-5Al_2_O_3_ sample by ^27^Al NMR. This amount might be too low to be detected by ^27^Al NMR. It is worthwhile to note that the H_2_ consumption of 5 nm In_2_O_3_ particles is substantially higher (0.35 mmol/g_cat_) when compared to the value of 0.042 mmol/g_cat_ found for 9 nm In_2_O_3_ particles also prepared by FSP.^17^ Normalized to the surface In content, this implies that ∼16% of the surface area of the 5 nm In_2_O_3_ particles is reduced, whereas this amounts to only 4% for 9 nm In_2_O_3_ particles. The higher reducibility of the smaller particles is in line with recent observations of enhanced reducibility of very small CeO_2_ particles of a few nanometers.^34^

The introduction of Al further increases the reducibility of the samples. This can be due to the destabilization of the In_2_O_3_ lattice by Al substitutions for In, which is evident at a low Al content. At higher Al content, there is also a contribution of decreasing In_2_O_3_ particle size due to the stabilizing effect of alumina domains. Nevertheless, as the particle size does not change significantly, it cannot be excluded that interfacial sites between In_2_O_3_ and Al_2_O_3_ also enhance the In_2_O_3_ reducibility. From the above, we conclude that Al as a dopant facilitates the formation of O_v_ in In_2_O_3_. At the same time, the decrease of the In_2_O_3_ particle size due to the stabilizing effect of Al_2_O_3_ at the surface and the interface between In_2_O_3_ and Al_2_O_3_ results in a more heterogeneous reduction of In_2_O_3_.

**Figure 6:**
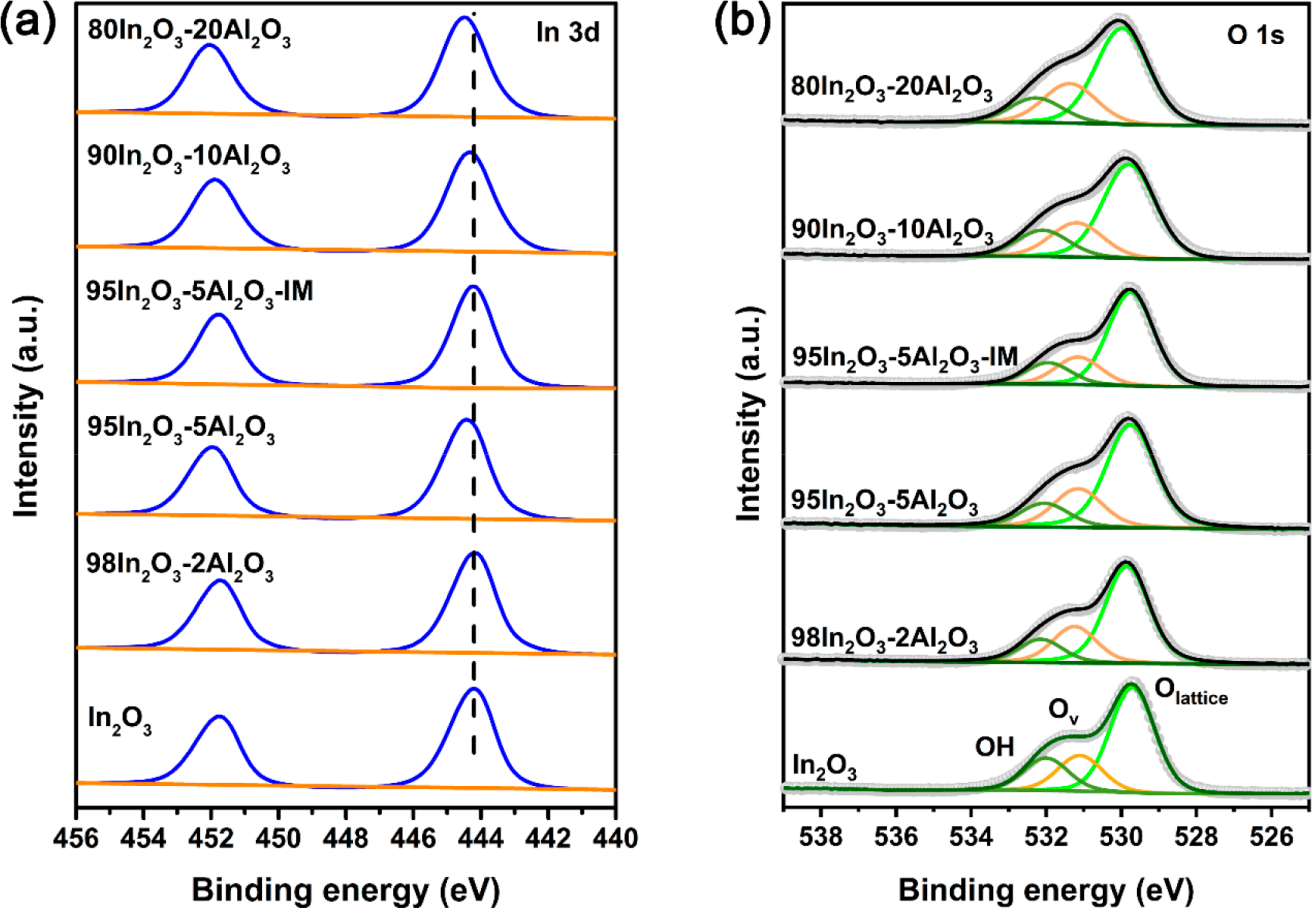
XPS spectra of the (a) In 3d and (b) O 1s regions of the as-prepared In_2_O_3_ and Al-doped In_2_O_3_ samples.

XPS was performed to determine the oxidation state of In in the as-prepared samples. Figure 6a shows the In 3d XPS spectra obtained for the In_2_O_3_–Al_2_O_3_ samples. The as-prepared In_2_O_3_ sample shows two peaks at 444.3 and 451.7 eV, which can be ascribed to the 3d_5/2_ and 3d_3/2_ states of In^3+^ (spin–orbital splitting of 7.5 eV).^35^ These features shift to higher binding energies in the Al-containing samples prepared by FSP. Such an effect is likely the result of lattice distortions, thus supporting the conclusion of Al doping in In_2_O_3_.^36^ The O 1s XPS spectra are shown in Figure 6b, where the spectral contributions at 530.3, 531.7, and 532.5 eV can be assigned to lattice O (O_lattice_), O atoms near oxygen vacancy (O_v_) sites, and surface hydroxyls (OH), respectively.^7^Figure S4 shows the C 1s and Al 2p XPS spectra of the In_2_O_3_–Al_2_O_3_ samples. The peaks at 284.8, 287.5, 288.5, and 289.1 eV in the C 1s XPS spectra represent contributions of C–C, C=O, O–C=O, and carbonate groups.^37,38^ Determining the O_v_ contribution from the O 1s XPS spectra is hampered by the overlap of the carbonate contribution due to CO_2_ adsorption on In_2_O_3_ and the Al 2p XPS peaks.^39,40^ To estimate the amount of O_v_ upon Al doping, we developed a procedure to separate Al 2p XPS contributions from the carbonate contribution in the O 1s spectra (Table S2). In this way, we established that the concentration of O_v_ exhibits a maximum concerning the Al content in FSP-derived catalysts. The samples with 2 and 5 mol % Al have the highest O_v_ concentration. We tentatively attribute this trend to predominant Al doping in In_2_O_3_ at low Al content, resulting in easier O_v_ formation, while segregated Al_2_O_3_ covers part of the In_2_O_3_ surface at high Al content. The formation of small Al_2_O_3_ domains can also explain the smaller In_2_O_3_ particle size observed by XRD, as Al_2_O_3_ stabilizes In_2_O_3_ particles. Additionally, the 95In_2_O_3_-5Al_2_O_3_-IM catalyst exhibits a higher fraction of lattice oxygen with a lower fraction of O_v_ when compared to the FSP-derived 95In_2_O_3_-5Al_2_O_3_ catalyst, which could be due to the coverage of In_2_O_3_ with small Al_2_O_3_ clusters as supported by textural analysis (Table 1). The surface elemental composition of the as-prepared Al-doped In_2_O_3_ samples, as determined by XPS, is given in Table S3. The atomic Al/In surface ratio in the as-prepared samples is similar to the bulk ratio, which suggests that there is no strong segregation into large Al_2_O_3_ domains in these samples.

### 3.3 Catalytic Performance

**Figure 7:**
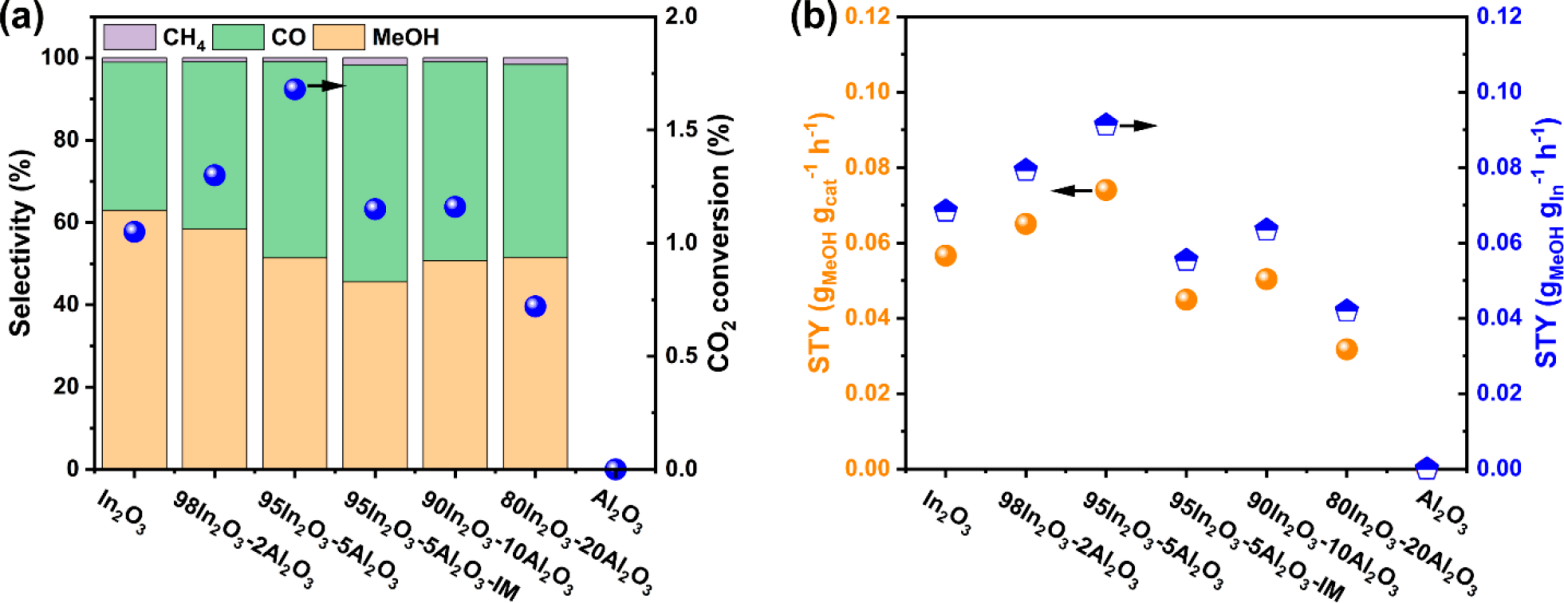
(a) CO_2_ conversion and methanol selectivity. (b) Space-time yield of methanol as a function of the Al content on In_2_O_3_. Reaction conditions: 260 °C, 3.0 MPa, GHSV = 30000 mL·h^–1^·g^–1^ with a feed (H_2_/CO_2_/N_2_ = 30/10/10) flow rate of 50 mL/min.

The catalytic performance of the as-prepared samples is shown in Figure 7. The catalysts were evaluated in CO_2_ hydrogenation at a temperature of 260 °C and a pressure of 30 bar. To compare the performance of the In_2_O_3_–Al_2_O_3_ catalysts, the CO_2_ conversion in all experiments was below 2%, which is well below the CO_2_ equilibrium conversion of ∼20% under the given conditions.^41^ The CO_2_ conversion exhibits a maximum with respect to the Al content. The highest conversion of 1.7% is obtained for the 95In_2_O_3_-5Al_2_O_3_ catalyst. Its activity is ∼60% higher than the activity of pure In_2_O_3_. Under the given reaction conditions, the methanol selectivity of In_2_O_3_ is 63%. The methanol selectivity was seen to decrease slightly with an increasing Al content. With an increase in Al loading to 20 mol %, the methanol selectivity decreased to 51%. The main other reaction product is CO, with minor amounts of CH_4_ being observed for all samples. Notably, CO_2_ was not converted on FSP-derived Al_2_O_3_. The methanol yield, expressed as the methanol space-time yield (STY), is plotted in Figure 7b and exhibits a maximum as a function of the Al content. The STY of 95In_2_O_3_-5Al_2_O_3_ amounts to 0.074 g_MeOH_ g_cat_^–1^ h^–1^, which compares favorably with the value of 0.057 g_MeOH_ g_cat_^–1^ h^–1^ for In_2_O_3_. Compared to these two samples, the STY of the impregnated sample 95In_2_O_3_-5Al_2_O_3_-IM is lower (0.045 g_MeOH_ g_cat_^–1^ h^–1^). This difference shows that the presence of Al_2_O_3_ on the In_2_O_3_ surface inhibits CO_2_ conversion and methanol formation, likely by covering part of the active sites of In_2_O_3_. Combined with the XRD and NMR results, the results in Figure 7 indicate that Al^3+^ doped in In_2_O_3_ can increase the O_v_ concentration, improving the CO_2_ conversion with respect to undoped In_2_O_3_. The samples with a relatively high Al content and the impregnated sample exhibit lower rates of methanol formation compared to pure In_2_O_3_, indicating that the presence of Al_2_O_3_ particles on the In_2_O_3_ surface blocks active sites for methanol formation. However, when normalized by surface area, In_2_O_3_ and 95In_2_O_3_-5Al_2_O_3_-IM show comparable methanol rates (Figure S5). It should be noted that, despite the lower surface area of the 95In_2_O_3_-5Al_2_O_3_-IM catalyst in comparison to In_2_O_3_, it exhibits a higher CO_2_ conversion but a lower methanol selectivity. These results suggest that impregnation of Al on the In_2_O_3_ surface decreases the number of active sites for methanol formation but increases the number for CO formation. It is speculated that the formation of CO is related to the presence of the Al_2_O_3_ domains that have interfacial sites with the In_2_O_3_. This would also agree with the increasing CO selectivity with Al content for the FSP samples. Figure S6 illustrates the performance of the samples with time on stream (TOS). While all catalysts exhibit a declining conversion with TOS, In_2_O_3_, 98In_2_O_3_-2Al_2_O_3_, and 95In_2_O_3_-5Al_2_O_3_ deactivate less strongly than 80In_2_O_3_-20Al_2_O_3_. The impregnated sample shows the smallest loss of catalytic activity among the catalysts evaluated.

### 3.4 Characterization of Used In_2_O_3_–Al_2_O_3_

**Figure 8:**
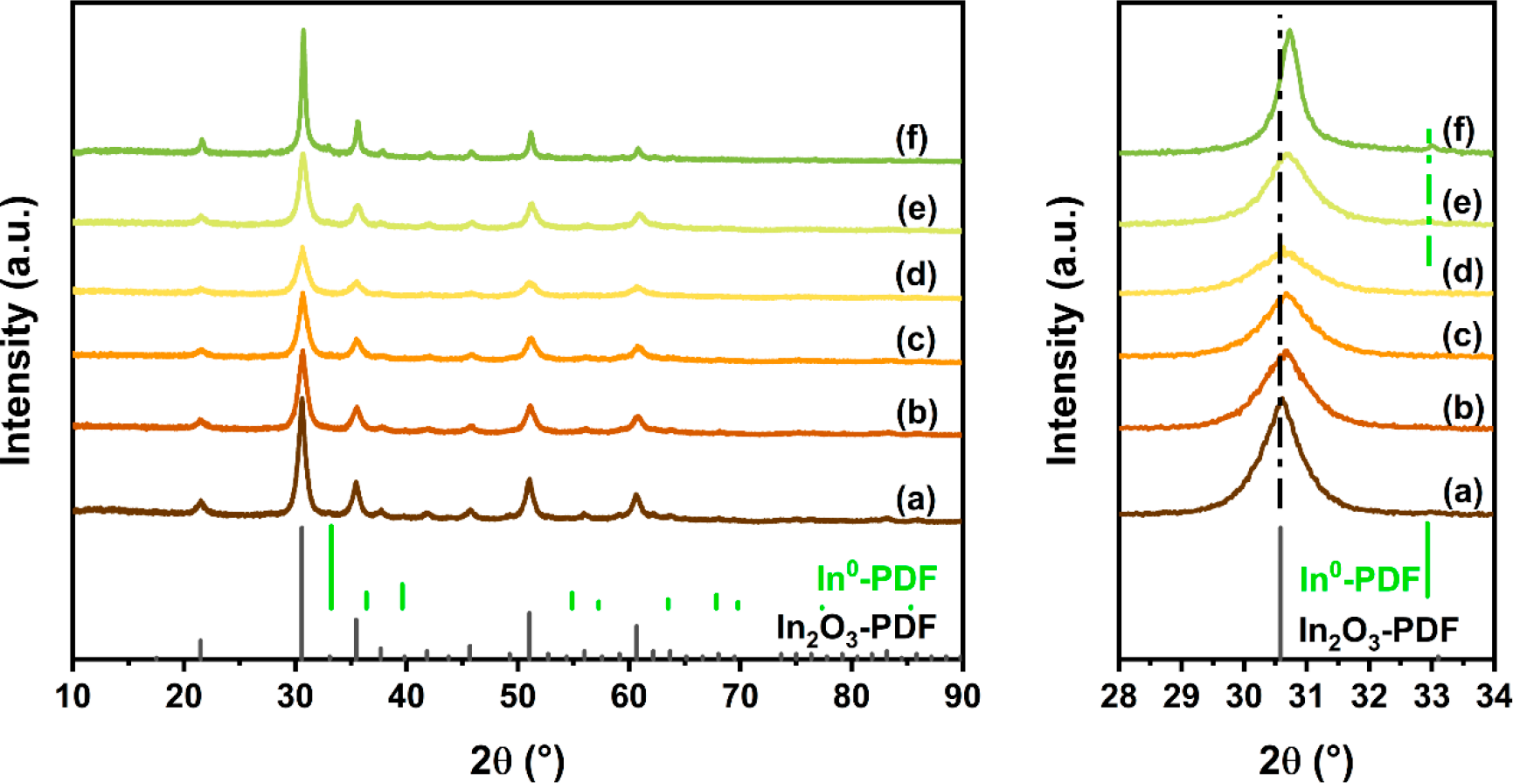
XRD patterns of used (a) In_2_O_3_, (b) 98In_2_O_3_-2Al_2_O_3_, (c) 95In_2_O_3_-5Al_2_O_3_, (d) 95In_2_O_3_-5Al_2_O_3_-IM, (e) 90In_2_O_3_-10Al_2_O_3_, and (f) 80In_2_O_3_-20Al_2_O_3_ samples.

**Figure 9:**
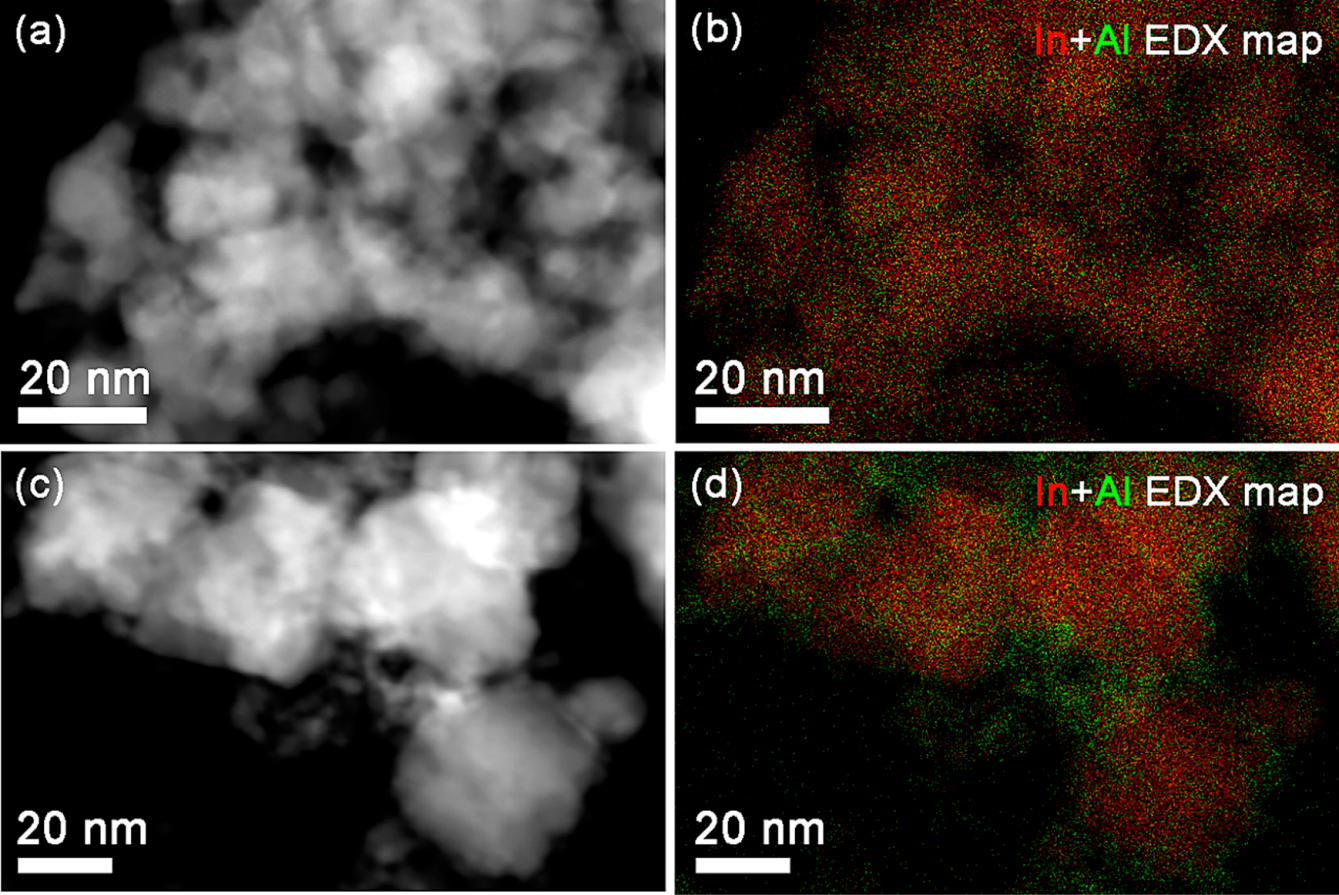
HAADF images and STEM-EDX maps of used (a), (b) 95In_2_O_3_-5Al_2_O_3_, and (c), (d) 80In_2_O_3_-20Al_2_O_3_ samples (In red, Al green).

The XRD patterns of the used catalysts are shown in Figure 8. The peak shifts observed for the used samples are comparable to those observed for the corresponding as-prepared samples. This indicates that the Al doping situation did not substantially change during the CO_2_ hydrogenation reaction. For the used 80In_2_O_3_-20Al_2_O_3_ catalyst, the additional diffraction line observed at ∼33° can be ascribed to metallic In. Judging from the XRD patterns, this sample contains a small amount of metallic In. The average size of the In_2_O_3_ particles in most of the used samples, as estimated by the Scherrer equation, is ∼9 nm, which means that the In_2_O_3_ particles sintered during the CO_2_ hydrogenation reaction (Table 1). The exception is used 80In_2_O_3_-20Al_2_O_3_, which contains substantially larger In_2_O_3_ particles (ca. 15 nm). The average size of the metallic In particles in the used 80In_2_O_3_-20Al_2_O_3_ is ca. 50 nm. The large size of metallic In is expected, given its low melting temperature.^42^ Notably, sintering is slightly less extensive for the used 95In_2_O_3_-5Al_2_O_3_-IM catalyst with an XRD particle size of 7.8 nm (Figure 8 and Table 1). In line with these XRD findings, TEM shows that the used 80In_2_O_3_-20Al_2_O_3_ catalyst is made up of larger particles than the other catalysts (Figure S7). The In_2_O_3_–Al_2_O_3_ catalysts used were also investigated by STEM-EDX (Figures 9 and S8). At an Al content of 5 mol %, Al remains homogeneously distributed in the FSP and impregnated samples. The EDX maps for 80In_2_O_3_-20Al_2_O_3_ show that Al is present as small particles of ca. 3 nm around the larger In_2_O_3_ particles. Overall, these data suggest that doping low amounts of Al did not significantly alter the crystal structure of In_2_O_3_. Both In_2_O_3_ and In_2_O_3_ doped with small amounts of Al exhibited similar degrees of sintering during the CO_2_ hydrogenation reaction. However, as the Al content increased, it led to the formation of smaller In_2_O_3_ particles, which were more prone to reduction during the CO_2_ hydrogenation reaction, as supported by the large H_2_ consumption observed during H_2_-TPR. This led to the formation of relatively large metallic In particles, which are probably also due to the high mobility of metallic In. Moreover, the smaller In_2_O_3_ particles initially stabilized by a higher Al content exhibit more pronounced sintering based on XRD observations.

**Figure 10:**
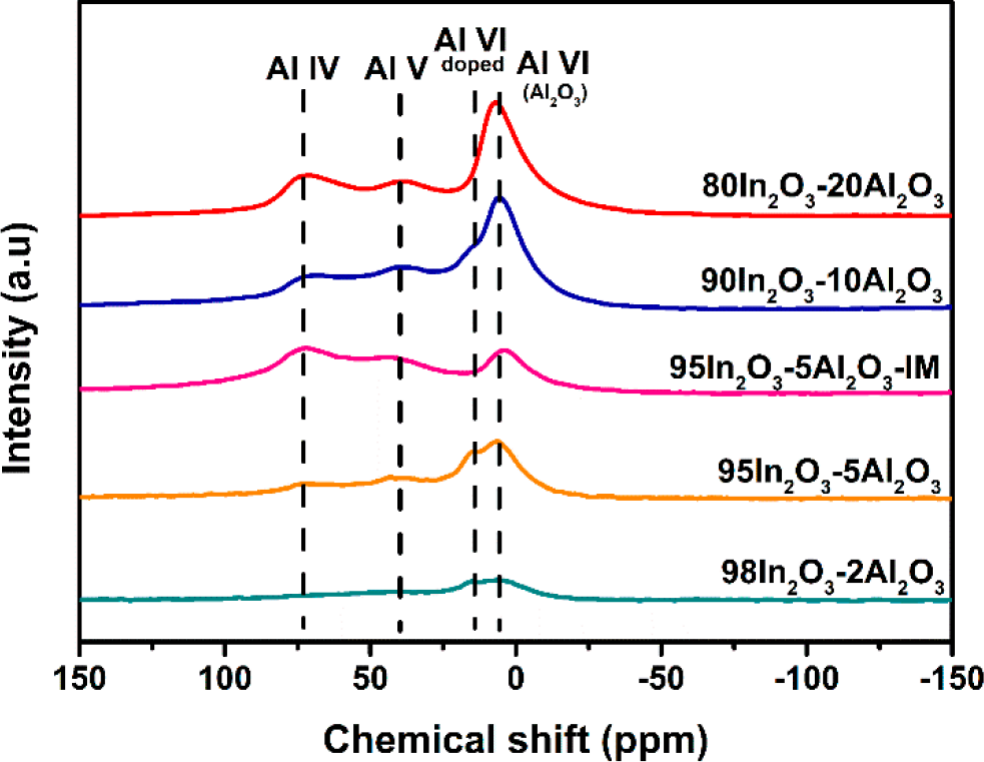
^27^Al NMR spectra of the used In_2_O_3_–Al_2_O_3_ samples.

The ^27^Al NMR spectra of the used catalysts are given in Figure 10, while the results of their deconvolution are listed in Table 2. The intensity and chemical shifts of the Al^IV^, Al^V^, and Al^VI^ features of the samples with a low Al content are comparable to those of the corresponding as-prepared In_2_O_3_–Al_2_O_3_ catalysts. Above 5 mol % Al, the absolute amount of Al^V^ increased, while the amount of doped Al^VI^ decreased compared to the corresponding as-prepared samples (Figure S2). This is most likely due to the migration of some of the doped Al species from the In_2_O_3_ lattice to the surface during the reaction.

The XPS spectra of the used samples are shown in Figure S9. Before XPS analysis, the samples were transferred from the reactor to a glovebox without contact with the ambient, where they were placed in an airtight transfer holder for transfer into the XPS apparatus. For all samples, the In 3d_5/2_ feature is located at 444.3 eV, which means that In is mainly present in the +3 state during the CO_2_ hydrogenation reaction. Even for the used 80In_2_O_3_–Al_2_O_3_ catalyst, XPS did not show the presence of reduced In, which may indicate the high reactivity of metallic In toward trace oxygen during sample transfer steps. Table S3 shows that, for the used samples containing more than 5 mol % Al, the Al-to-In ratio is higher than that of the corresponding as-prepared catalysts. This observation indicates an accumulation of Al species on the catalyst surface, which may hint at the migration of doped Al species during the CO_2_ hydrogenation reaction. This is consistent with the NMR results. The highest Al-to-In ratio is obtained for the used 80In_2_O_3_-20Al_2_O_3_ catalyst, with a value nearly two times higher than that of the corresponding as-prepared sample. For the samples containing 2 and 5 mol % Al, the sintering of Al_2_O_3_ is much less significant.

### 3.5 Promoting Role of Al

**Figure 11:**
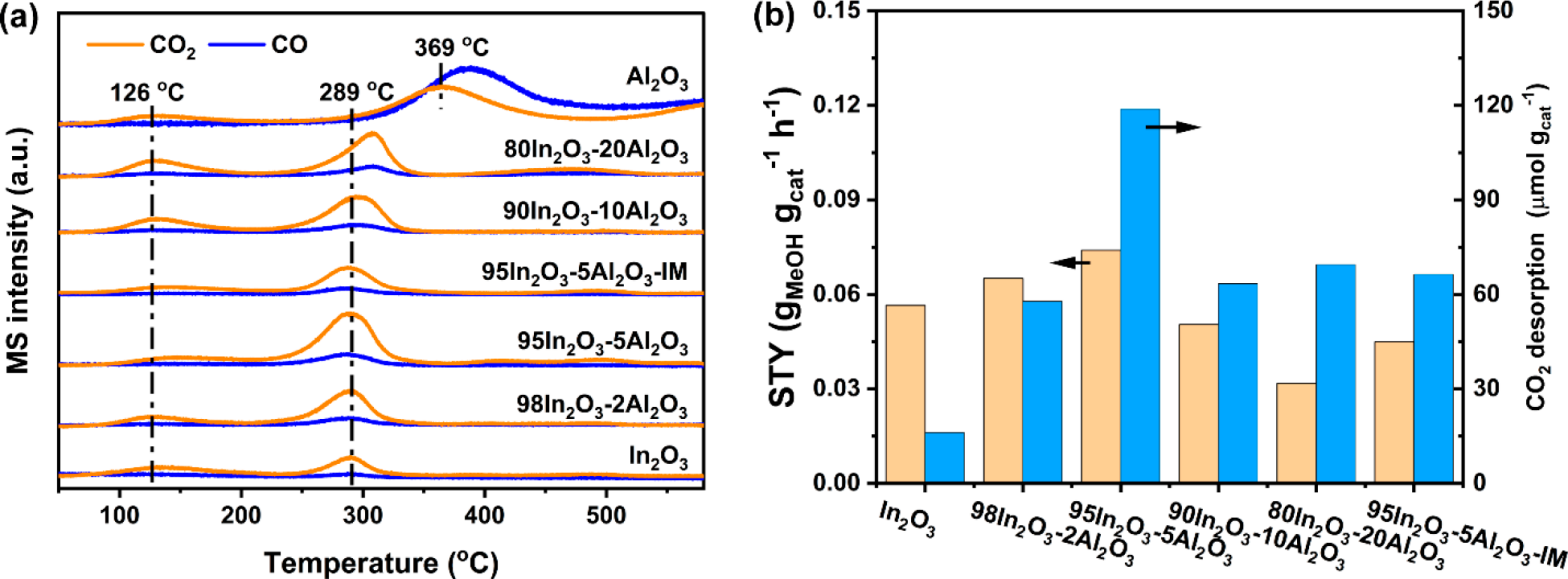
(a) CO_2_-TPD profiles of In_2_O_3_–Al_2_O_3_ catalysts with different Al content and Al_2_O_3_. (b) The relation between methanol space-time yield (STY) over the In_2_O_3_–Al_2_O_3_ catalysts during CO_2_ hydrogenation (Figure 7) and the amount of CO_2_ desorbed, which was obtained by quantifying the TPD desorption peak in the range of 200 to 350 °C (Table S1).

To understand the influence of Al on the adsorption and activation of CO_2_, we performed CO_2_-TPD experiments. Before CO_2_-TPD, the catalysts were used in CO_2_ hydrogenation (260 °C, 30 bar, CO_2_/H_2_/N_2_ = 10/30/10 mL/min) for 14 h and transferred without air exposure to the TPD setup. Then, the catalyst was exposed to aqueous CO_2_ for 2 h at 50 °C. Figure 11 shows the subsequently obtained TPD profiles in flowing He. The desorption peak at ∼120 °C can be ascribed to weakly adsorbed CO_2_, while the feature between 200 and 350 °C corresponds to chemisorbed CO_2_ on O_v_ sites.^43^ The intensity of the low-temperature feature due to weakly adsorbed CO_2_ is comparable between the samples with 2 and 5 mol % Al content and In_2_O_3_. This feature is slightly stronger for the 90In_2_O_3_-10Al_2_O_3_ and 80In_2_O_3_-20Al_2_O_3_ catalysts. We speculate that the additional weakly adsorbed CO_2_ on these samples stems from the interaction of the CO_2_ with Al_2_O_3_ domains dispersed on the In_2_O_3_ surface, which are present at an Al loading exceeding 5 mol %. Notably, the CO_2_ desorption peak position shifts to a slightly higher temperature for these samples, which indicates the involvement of other sites. To confirm that the CO_2_ desorption peak between 200 and 350 °C is due to CO_2_ adsorbed on the O_v_ sites, we also carried out the CO_2_-TPD experiments after treating the 95In_2_O_3_-5Al_2_O_3_ catalyst in an O_2_-containing flow, which should oxidize the O_v_ (Figure S10). The resulting TPD profile after CO_2_ exposure did not contain the medium-temperature feature, confirming the role of O_v_ in CO_2_ adsorption. Additionally, we observed a large CO desorption peak at 360 °C during the CO_2_-TPD of Al_2_O_3_, indicating that CO_2_ adsorbed on Al_2_O_3_ can be decomposed into CO at elevated temperature.

The amount of CO_2_ adsorbed on O_v_ in the In_2_O_3_–Al_2_O_3_ catalysts was quantified based on the CO_2_-TPD peak area between 200 and 350 °C. The CO_2_ signal was calibrated using the thermal decomposition of MgCO_3_.^44^ For quantification, we used a calibration curve correlating the amount of CO_2_ desorbed to the peak area (*m*/*z* = 44), as shown in Figure S11. The amount of CO_2_ desorption increases from 16.1 μmol/g_cat_ for In_2_O_3_ to the highest value of 118.8 μmol/g_cat_ for 95In_2_O_3_-5Al_2_O_3_, followed by a decrease to 63.4 μmol/g_cat_ for 80In_2_O_3_-20Al_2_O_3_ (Table S1). The highest O_v_ concentration for the 95In_2_O_3_-5Al_2_O_3_ sample can be correlated to the highest methanol STY. The impregnated 95In_2_O_3_-5Al_2_O_3_-IM sample with the same nominal Al content exhibits a similar CO_2_ desorption feature, yielding a much lower O_v_ concentration of 66.4 μmol/g_cat_. The 90In_2_O_3_-10Al_2_O_3_ and 80In_2_O_3_-20Al_2_O_3_ catalysts show comparable O_v_ concentrations as 95In_2_O_3_-5Al_2_O_3_-IM. It should be noted that the amount of CO_2_ desorbed during the TPD experiment is significantly less than the surface reducibility determined by H_2_-TPR (Table S1). The H_2_-TPR data likely overestimate the O_v_ concentration, as it was obtained by reduction up to a higher temperature than the reaction temperature in the absence of CO_2_, which can act as an oxidant. On the other hand, it is also likely that O removal from the surface will lead to surface restructuring and even In metal formation,^21,45^ which renders it very challenging to determine the concentration of O_v_ for these samples. The weak correlation between the methanol formation rates and the amount of chemisorbed CO_2_ (integrated peak from the CO_2_-TPD between 200 to 350 °C) is shown in Figure 11b. Specifically, when the Al content is below 5 mol %, the amount of CO_2_ desorption and methanol rates increase with Al loading, while increasing the Al content lowers both the amount of CO_2_ desorbed and the methanol rate. We also noticed an increase in the CO desorption peak upon the introduction of Al into In_2_O_3_ (Figure 11a and Table S1). This is most likely due to the role of carbonate species formed on Al_2_O_3_ domains that cover the active sites of In_2_O_3_. Although Al_2_O_3_ is not catalytically active, the CO_2_-TPD experiment of Al_2_O_3_ shows that the CO formation can occur already below 300 °C for Al_2_O_3_. Thus, the much higher dispersed Al_2_O_3_ domains in the Al-containing In_2_O_3_ can also contribute to the increased level of CO formation during CO_2_-TPD and CO_2_ hydrogenation. This aspect will be further investigated below by IR spectroscopy. This can also explain the finding that the impregnated reference sample with an Al content of 5 mol % shows a lower methanol rate despite adsorbing more CO_2_ than In_2_O_3_.

**Figure 12:**
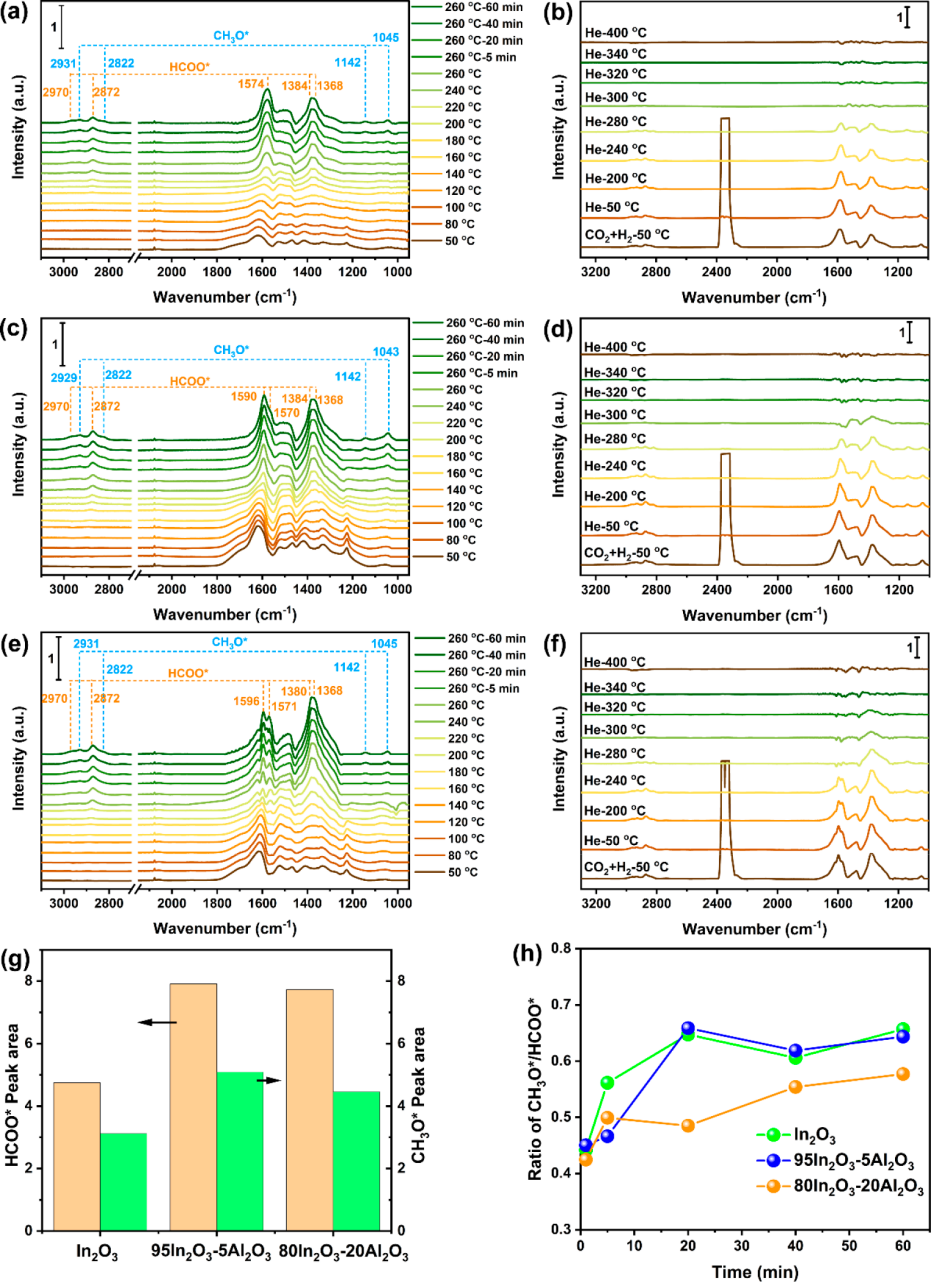
IR spectra during CO_2_ hydrogenation over (a) In_2_O_3_, (c) 95In_2_O_3_-5Al_2_O_3_, and (e) 80In_2_O_3_-20Al_2_O_3_. IR spectra after switching the reaction gas to He (1 bar) at 50 °C and rising temperature from 50 to 400 °C over (b) In_2_O_3_, (d) 95In_2_O_3_-5Al_2_O_3_, and (f) 80In_2_O_3_-20Al_2_O_3_. (g) Comparison of the peak area of HCOO* and CH_3_O* collected from the spectra at 260 °C under a reaction atmosphere for 60 min over In_2_O_3_, 95In_2_O_3_-5Al_2_O_3_, and 80In_2_O_3_-20Al_2_O_3_. (h) Evolution of the CH_3_O*/HCOO* ratio for In_2_O_3_, 95In_2_O_3_-5Al_2_O_3_, and 80In_2_O_3_-20Al_2_O_3_. Reaction conditions: CO_2_:H_2_ = 1:3, gas flow rate = 50 mL/min, and *P* = 10 bar. The intensity of the IR spectra is normalized by the weight of the catalyst pellet.

We investigated the surface species present on the various samples during temperature-programmed hydrogenation of CO_2_ by in situ IR spectroscopy. Figure 12a shows the relevant IR spectra for In_2_O_3_. At a temperature of 50 °C, exposure of In_2_O_3_ to a reactant mixture (H_2_/CO_2_ = 3, 10 bar, 50 mL/min) led to the formation of bicarbonate (HCO_3_^–^), monodentate carbonate (m-CO_3_^2–^), and bidentate carbonate (b-CO_3_^2–^) species as follows from bands at 1613 and 1225 cm^–1^ due to HCO_3_^–^, at 1422 and 1335 cm^–1^ due to m-CO_3_^2–^,^46^ and at 1633 and 1528 cm^–1^ due to b-CO_3_^2–^.^25^ An increase of the temperature to 200 °C led to the appearance of bands at 2970, 2872, 1574, 1384, and 1368 cm^–1^, which respectively are due to δ(CH) + *v*_s_(OCO), *v*(CH), *v*_as_(OCO), δ(CH), and *v*_s_(OCO) of formate (HCOO*).^25,47^ Further increasing the temperature to 260 °C results in the appearance of bands at 1045, 1142, 2822, and 2931 cm^–1^ due to adsorbed methoxy (CH_3_O*).^48,49^ Simultaneously, the formation of polycarbonates (p-CO_3_^2–^) was observed, indicated by the band at 1484 cm^–1^. We also studied the changes in the IR spectra by a subsequent switch of the feed gas from CO_2_ + H_2_ (H_2_/CO_2_ = 3, 10 bar, 50 mL/min) to pure H_2_ (1 bar, 50 mL/min) at 260 °C (Figure S12). The transition of the reaction from a semisteady state to a state where intermediates are removed from the surface results in a decrease in formate bands and an increase in methoxy bands. As such, this supports the mechanism that formate is the key intermediate for methoxy and methanol formation, which is in line with DFT calculations for In_2_O_3_.^50,51^

The IR spectra obtained during temperature-programmed CO_2_ hydrogenation on the 95In_2_O_3_-5Al_2_O_3_ catalyst indicate that the formation of HCOO* starts at a temperature of 180 °C lower than that of In_2_O_3_ (Figure 12c). The *v*_as_(OCO) band of HCOO* is shifted to a slightly higher wavenumber of 1590 cm^–1^ compared to In_2_O_3_. A shoulder is present at 1570 cm^–1^, which can be attributed to HCOO* on In_2_O_3_.^47^ As a reference, we also carried out similar temperature-programmed IR measurements of the inactive FSP-derived Al_2_O_3_ sample (Figure S13), which exhibited three broad bands at 1645, 1510, and 1404 cm^–1^ due to bicarbonate, b-CO_3_^2–^, and m-CO_3_^2–^ species, respectively. The absence of bands due to HCOO* or CH_3_O* indicates that CO_2_ adsorbed on Al_2_O_3_ can be converted to carbonates, which are not involved in catalytic reactions under the given conditions. Therefore, it can be speculated that the HCOO* band at 1590 cm^–1^ can be attributed to formate adsorbed on In sites close to the highly dispersed Al. The modification of In_2_O_3_ with ZrO_2_ in In_2_O_3_/ZrO_2_ and Co with ZrO_2_ in Co/ZrO_2_ led to similar shifts, which were explained by the presence of interfacial sites.^47,52^

The sample with 20 mol % Al contained more carbonates than In_2_O_3_ and 95In_2_O_3_-5Al_2_O_3_, as follows from the more intense bands at 1633, 1422, and 1335 cm^–1^ (Figure 12e). We attribute this to the presence of well-dispersed Al_2_O_3_ domains on the In_2_O_3_ surface. The *v*_as_(OCO) bands of HCOO* were observed at 1596 and 1571 cm^–1^ for 80In_2_O_3_-20Al_2_O_3_, suggesting that the surface contains both interfacial In–Al sites and unperturbed In sites.

The amount of HCOO* and CH_3_O* species was estimated by integrating the areas of the bands at 2872 and 2931 cm^–1^, respectively, under reaction conditions (260 °C, 10 bar, 60 min) for In_2_O_3_, 95In_2_O_3_-5Al_2_O_3_, and 80In_2_O_3_-20Al_2_O_3_ (Figure 12g). The presence of Al in In_2_O_3_ increases the amount of HCOO* and CH_3_O* species. Among samples investigated, the 95In_2_O_3_-5Al_2_O_3_ sample contains the most HCOO* and CH_3_O* species during the IR measurements, which is consistent with the highest methanol rates observed during catalytic CO_2_ hydrogenation. It should be noted that the in situ IR measurements were carried out at 10 bar, which is lower than the pressure used in the catalytic tests. Higher pressure typically leads to a higher rate of formate hydrogenation to methanol, explaining the preference in catalytic performance measurements for high pressure to increase the methanol selectivity. Combined with the CO_2_ absorption data (Figure 11 and Table S1), we infer that doping Al can increase the concentration of O_v_, representing active sites for the conversion of adsorbed CO_2_ into HCOO* intermediates relevant to methanol formation. The evolution of the CH_3_O*/HCOO* ratio during the exposure to the reactant mixture (H_2_/CO_2_ = 3, 10 bar) for In_2_O_3_, 95In_2_O_3_-5Al_2_O_3_, and 80In_2_O_3_-20Al_2_O_3_ is also shown in Figure 12. Notably, the 80In_2_O_3_-20Al_2_O_3_ sample exhibited a slower increase of the CH_3_O/HCOO* ratio toward a steady-state value compared to In_2_O_3_ and 95In_2_O_3_-5Al_2_O_3_. Moreover, the CH_3_O*/HCOO* ratio reaches a steady-state value lower than that of the other samples. These differences indicate the slower hydrogenation of HCOO* to CH_3_O* for this sample, despite the more intense HCOO* and CH_3_O* bands compared to those of In_2_O_3_ (Figure 12g). In addition, the catalytic data show that 95In_2_O_3_-5Al_2_O_3_ and 80In_2_O_3_-20Al_2_O_3_ exhibit similar methanol selectivity, which is however lower than the methanol selectivity for In_2_O_3_ (Figure 7). Based on the CO_2_-TPD and IR spectroscopy of Al_2_O_3_, we can conclude that CO_2_ adsorption leads to carbonate species, which can be decomposed into CO. Accordingly, we speculate that the increased CO selectivity in In_2_O_3_ upon introduction of Al can be attributed to the decomposition of HCOO*, CH_3_O*, and carbonate-type species formed during CO_2_ hydrogenation. For In_*x*_/ZrO_2_, it was observed that a low In loading on ZrO_2_ leads to a lack of dissociated H species, favoring the formation of CO.^47^ Similarly, the presence of Al_2_O_3_ domains in Al-containing In_2_O_3_ prepared by FSP results in a lower hydrogenation rate compared to that of In_2_O_3_, which can explain the formation of CO and the lower methanol selectivity.

To gain further insight into the relationship between the CO_2_ species probed during CO_2_-TPD and the rate of methanol formation, we carried out an operando IR measurement followed by temperature-programmed desorption in He (Figure 12b, d, and f). Upon switching the gas flow from the reaction mixture (CO_2_:H_2_ = 1:3, 10 bar, 50 mL/min) to He (100 mL/min, 1 bar) at 50 °C, the intense band due to gaseous CO_2_ disappeared from the IR spectrum of In_2_O_3_. Increasing the temperature to 280 °C led to the disappearance of bands of surface intermediates such as p-CO_3_^2–^ (1484 cm^–1^), HCOO* (2970, 2872, 1574, 1384, and 1368 cm^–1^), and CH_3_O* (1045, 1142, 2822, and 2931 cm^–1^). The CO_2_-TPD data (Figure 11a and Table S1) indicate that this is due to CO_2_ desorption and conversion of these intermediates to CO. It is worth noting that the bands related to gaseous CO were not detected. This can be attributed to the relatively high gas flow rate used in the IR measurements and the large gas volume of the cell compared with the amount of sample. Compared with In_2_O_3_, the bands characteristic for HCOO* and CH_3_O* disappear at the same temperature for 95In_2_O_3_-5Al_2_O_3_, whereas those for p-CO_3_^2–^ and m-CO_3_^2–^ species erode at a higher temperature of 300 °C. These carbonate species are even stronger retained on 80In_2_O_3_-20Al_2_O_3_, requiring a temperature of 320 °C for their decomposition. These observations indicate that the carbonates involving the Al_2_O_3_ domains are more stable than those adsorbed on In_2_O_3_. This can explain the shift in the CO_2_ desorption peak to higher temperatures of 90In_2_O_3_-10Al_2_O_3_ and 80In_2_O_3_-20Al_2_O_3_ catalysts compared to In_2_O_3_. The finding that carbonates are stable up to 320 °C, which is higher than the typical reaction temperatures employed, implies that carbonates may block the active sites for the hydrogenation of CO_2_ to methanol. Such an adverse effect of carbonates has been reported before.^53,54^ Nevertheless, the CO_2_-TPD shows that these carbonates already can start decomposing at a lower temperature. Overall, the formation of Al_2_O_3_ domains on In_2_O_3_ at high Al content contributes to decreased CO_2_ adsorption and conversion to methanol compared to In_2_O_3_ and increased formation of undesired CO.

### 3.6 Discussion

**Scheme 1:**
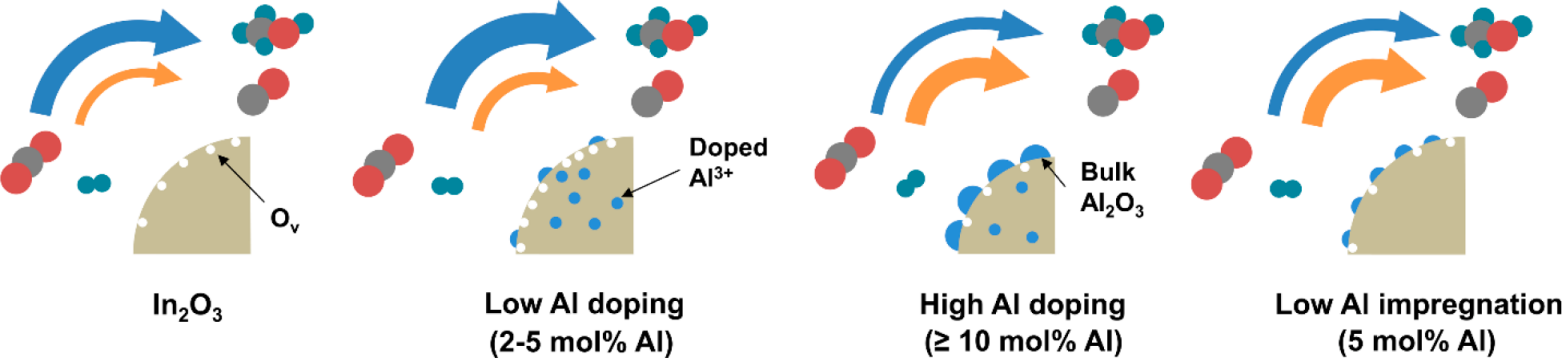
Schematic Representation of the Evolution of the Bulk and Surface Structure of In_2_O_3_ upon Introduction of Al by FSP and Impregnation The arrows qualitatively indicate the rate of the two main reactions, namely CO_2_ hydrogenation to methanol (blue) and the reverse water–gas shift reaction (orange).

Characterization of the Al-doped In_2_O_3_ samples shows that Al can be present as doped ions in the lattice of In_2_O_3_ and as dispersed Al_2_O_3_ domains on the In_2_O_3_ surface. The different roles of the Al species in methanol synthesis are illustrated in Scheme 1. According to NMR, the fraction of Al species in doped positions is lower than 30%, with the highest absolute amount of doped Al being ∼1.2 mol %. This implies that all Al-containing samples also contain Al species at the In_2_O_3_ surfaces, likely present as small Al_2_O_3_ patches. The correlation between the number of O_v_ and the number of doped Al suggests that doping Al into the In_2_O_3_ lattice facilitates the removal of the surface O atom. This is likely an intrinsic effect of the presence of Al in the In_2_O_3_ lattice, as the impact of the particle size can be excluded. At low Al content (0–5 mol %) where the O_v_ concentration strongly increased, the particle sizes and surface areas of the used In_2_O_3_–Al_2_O_3_ samples were comparable. As the Al–O bond is significantly stronger (511 kJ/mol) than the In–O bond (346 kJ/mol),^55,56^ it is thus likely that the lattice distortions due to substitution of In^3+^ with the smaller Al^3+^ ion cause the higher reducibility. Similar results were reported for the Ni/In_2_O_3_ catalyst.^17^ The increased O_v_ concentration due to Al doping results in a larger amount of adsorbed CO_2_, which can be hydrogenated to formate, methoxy, and methanol, explaining the higher methanol reaction rate compared to In_2_O_3_. At higher Al content (>5 mol %), the stabilizing effect of the Al_2_O_3_ domains on In_2_O_3_ is evident from a decrease of the size of the In_2_O_3_ particles in the as-prepared samples. However, this goes together with significant agglomeration of In_2_O_3_ during CO_2_ hydrogenation, while also the formation of relatively large metallic In particles was observed. We speculate that the smaller In_2_O_3_ particles can be more easily reduced, resulting in strong agglomeration of In_2_O_3_ and the formation of metallic In, which is prone to sintering given the low melting temperature of metallic In.^45^ In line with this, the sintering of the slightly larger In_2_O_3_ particles that were impregnated with Al did not grow as much in size as did the FSP samples. The increasing coverage of the active In_2_O_3_ surface with Al_2_O_3_ results in a lower methanol reaction rate, the optimum performance being observed for the 95In_2_O_3_-5Al_2_O_3_ catalyst. When In_2_O_3_ was impregnated with the same amount of Al, the methanol reaction rate was significantly lower, which can be attributed to the absence of doped Al and the more significant coverage of In_2_O_3_ with Al_2_O_3_. The presence of Al_2_O_3_ also results in a decrease in the methanol selectivity due to a higher rate of the rWGS reaction. This is most likely caused by the decomposition of carbonates formed on the Al_2_O_3_ domains or their interfaces with In_2_O_3_. Another factor that can contribute to higher CO selectivity is suppression of H_2_ activation. Various DFT studies show that the In_2_O_3_ surface can heterolytic dissociate H_2_,^15,57^ which results in different H species relevant to the hydrogenation of CO_2_ to formate, followed by hydrogenation to methoxy and methanol. H_2_ activation is much more difficult on Al_2_O_3_,^58^ which can explain a shift from methanol to CO with increasing Al_2_O_3_ coverage of the In_2_O_3_ surface together with the decreasing CO_2_ conversion rate.

We also compared the performance of these In_2_O_3_–Al_2_O_3_ catalysts with other metal oxide-promoted In_2_O_3_ catalysts, specifically In_2_O_3_–ZrO_2_. The introduction of In_2_O_3_ (9.0 wt % In) on ZrO_2_ led to an order of magnitude higher methanol yield compared to In_2_O_3_, which is clearly a better result than achieved here by the addition of Al.^7^ Zr plays different promoting roles, such as acting as a structural promoter, enhancing oxygen vacancy (O_v_) production, and contributing to the construction of the active sites.^21,59,60^ The role of doped Al is likely limited to creating more of the O_v_ sites in In_2_O_3_ with a less prominent role as structural promoter given the superior hydrothermal stability of ZrO_2_ compared to Al_2_O_3_.

## 4 Conclusions

In this work, a series of In_2_O_3_–Al_2_O_3_ samples were prepared by using one-step FSP and impregnation methods and evaluated for their catalytic performance in CO_2_ hydrogenation to methanol. The 95In_2_O_3_-5Al_2_O_3_ sample (5 mol % Al) exhibited the highest methanol yield, which is ca. 30% higher than unpromoted In_2_O_3_. Using TPR, XPS, CO_2_-TPD, and IR and ^27^Al MQMAS NMR spectroscopy, it was established that doped Al^3+^ sites facilitate the formation of surface oxygen vacancies on In_2_O_3_, which are involved in CO_2_ adsorption and subsequent hydrogenation to formate, methoxy, and, finally, methanol. CO is observed as a side-product of the unselective decomposition of surface intermediates in all samples. Only a small amount of ca. 1.2 mol % can dissolve in the In_2_O_3_ lattice, implying that Al species are also present at the In_2_O_3_ surface. These form small Al_2_O_3_ domains that cover the surface. At higher Al content, these domains lower the activity by blocking active sites and adsorb CO_2_ as carbonates that under catalytic conditions contribute to CO formation through carbonate decomposition and suppress hydrogen activation. The In_2_O_3_ particles grow during the CO_2_ hydrogenation reaction. The sintering of In_2_O_3_ particles was limited at a low Al content. However, the slightly smaller In_2_O_3_ particles in the presence of more Al_2_O_3_ domains agglomerated more extensively, which is likely due to the high reducibility of the smaller In_2_O_3_ particles. Concomitantly, this led to significant formation of metallic In particles. These findings show how a small amount of Al introduced into the In_2_O_3_ lattice through FSP can improve the methanol yield due to the formation of more oxygen vacancies. Excess Al, however, decreases the performance through blocking of active sites by Al_2_O_3_ domains. Subtle particle size effects of In_2_O_3_ are also noted, where particles that are too small are prone to agglomeration.

## Supporting Information Available

XRD patterns for Al_2_O_3_; Correlation between Al content and fraction of Al species determined by ^27^Al NMR; Comparison of catalytic performance normalized by the surface area of the catalysts; Space-time yield of methanol along with the time on stream over the In_2_O_3_–Al_2_O_3_ catalysts; XPS spectra and TEM results of the In_2_O_3_–Al_2_O_3_ catalysts; CO_2_-TPD profiles of the 95In_2_O_3_-5Al_2_O_3_ catalyst with O_2_ pretreatment; TPD profiles of MgCO_3_ and a calibration curve correlating the amount of CO_2_ desorbed to the peak area; In situ IR spectra recorded after CO_2_ hydrogenation and subsequent H_2_ reduction over In_2_O_3_; Tables of data and elemental composition (PDF)
