# Supplementary material for: Al Promotion of In2O3 for CO2 Hydrogenation to Methanol
Source: ACS Catal. 2023 Nov 22;13(24):15730–45. doi: 10.1021/acscatal.3c04620 (PMC10728901; doi:10.1021/acscatal.3c04620)
Supplement: Supplementary file 1 — cs3c04620_si_001.pdf [file cs3c04620_si_001.pdf]

Supporting Information

Al Promotion of In_2O_3 for CO_2 Hydrogenation to Methanol

*Liang Liu, Brahim Mezari, Nikolay Kosinov, and Emiel J.M. Hensen**

Laboratory of Inorganic Materials and Catalysis, Department of Chemical Engineering and Chemistry, Eindhoven University of Technology, P.O. Box 513, 5600 MB Eindhoven, The Netherlands

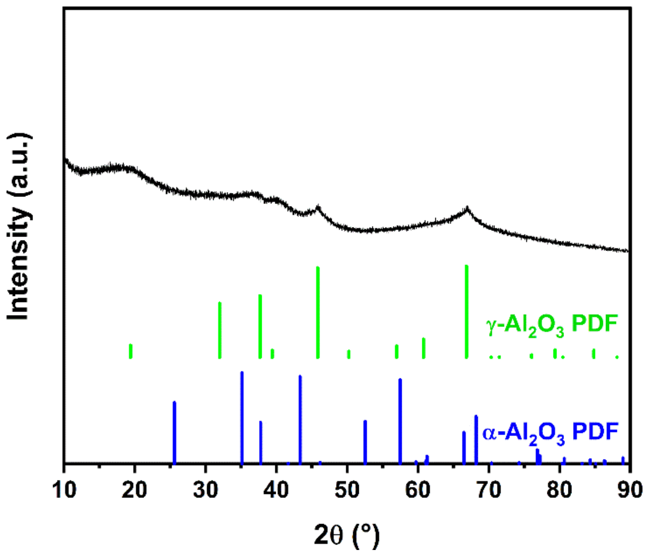

Figure S1. XRD pattern of Al_2O_3 prepared by FSP.

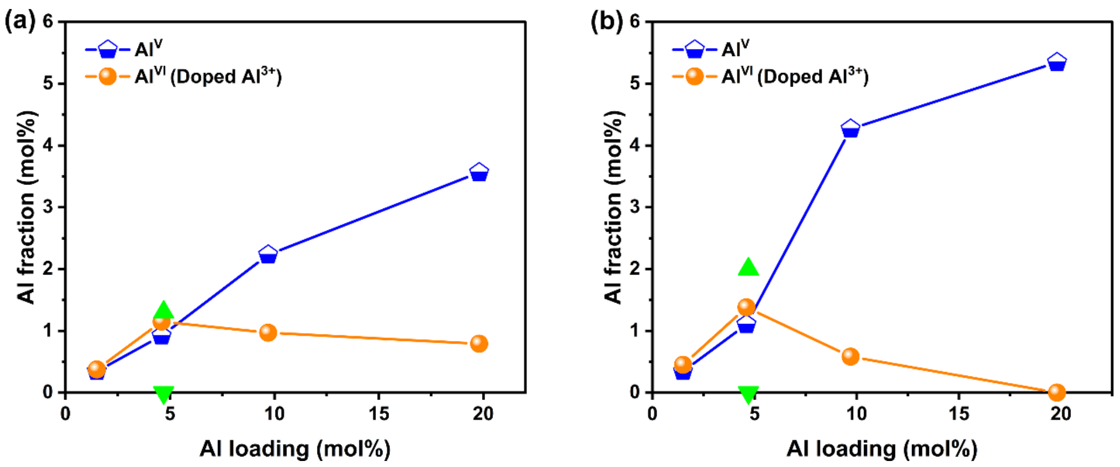

Figure S2. Relation between the amount of Al species as observed by ^{27}Al NMR spectroscopy and the Al content of In_2O_3-Al_2O_3 samples prepared by FSP (a) before and (b) after CO_2 hydrogenation reaction (260 °C, 30 bar, $CO_2/H_2/N_2$ = 10/30/10 mL/min, 14 h). The amount of Al^V (▲) and doped Al^{VI} (▼) species in 95In_2O_3-5Al_2O_3-IM catalyst is shown.

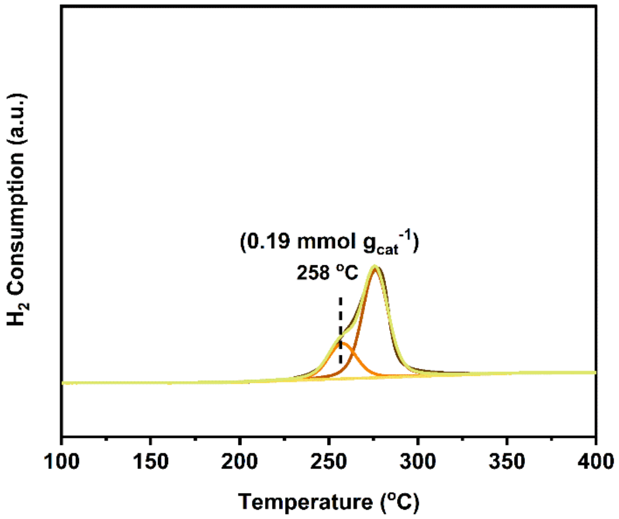

Figure S3. Deconvolution of H_2-TPR profile for $95In_2O_3$-$5Al_2O_3$-IM sample.

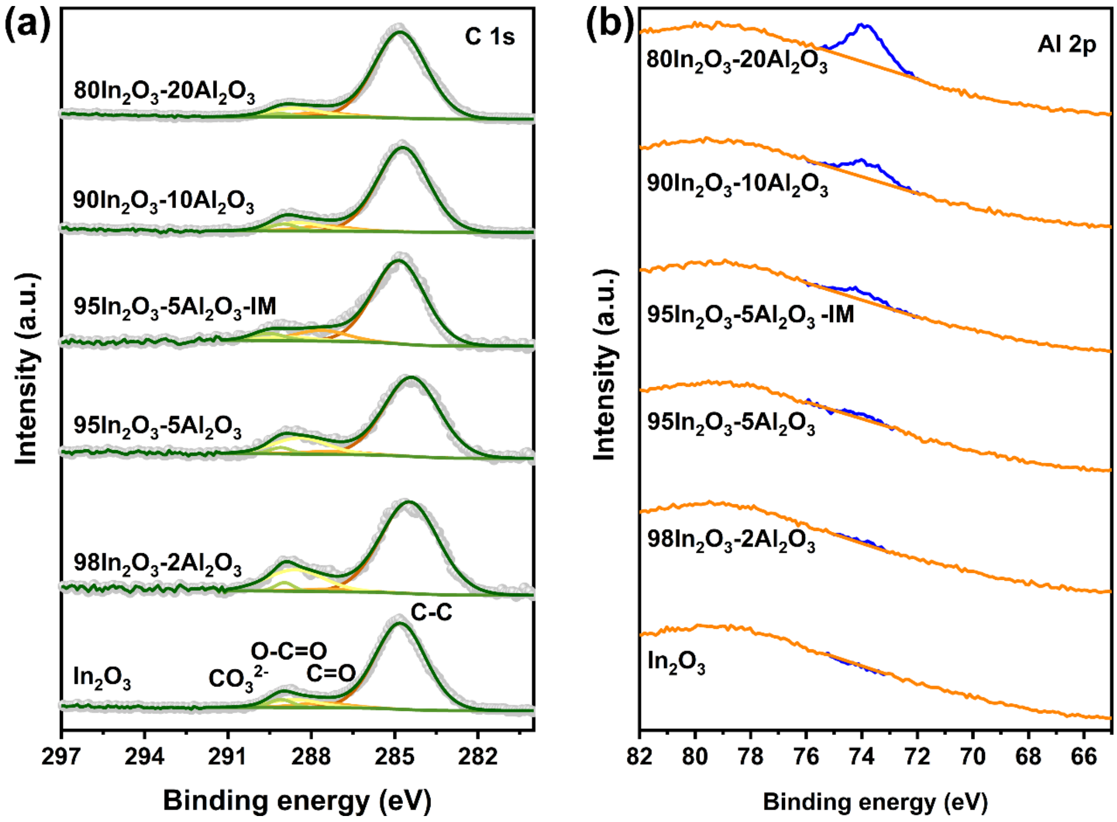

Figure S4. XPS spectra of the C 1s and Al 2p signals recorded for as-prepared In_2O_3-Al_2O_3 samples.

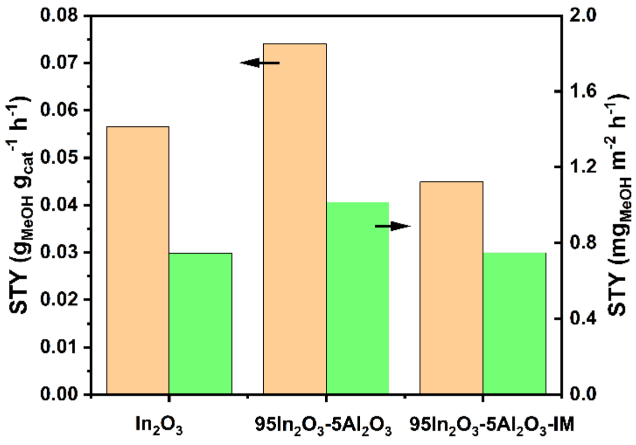

Figure S5. Space-time yield of methanol over In_2O_3, $95In_2O_3$-$5Al_2O_3$ and $95In_2O_3$-$5Al_2O_3$-IM catalysts. Reaction conditions: 260 °C, 3.0 MPa, GHSV = 30000 mL·h^{-1}·g^{-1} with a feed ($H_2/CO_2/N_2$=30/10/10) flow rate of 50 mL/min.

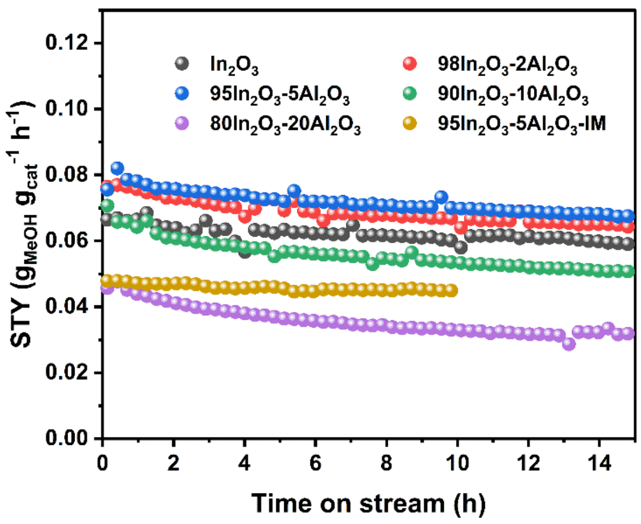

Figure S6. Space-time yield of methanol along with the time on stream over the In_2O_3-Al_2O_3 catalysts. Reaction conditions: 260 °C, 3.0 MPa, GHSV = 30000 mL·h^{-1}·g^{-1} with a feed ($H_2/CO_2/N_2$=30/10/10) flow rate of 50 mL/min.

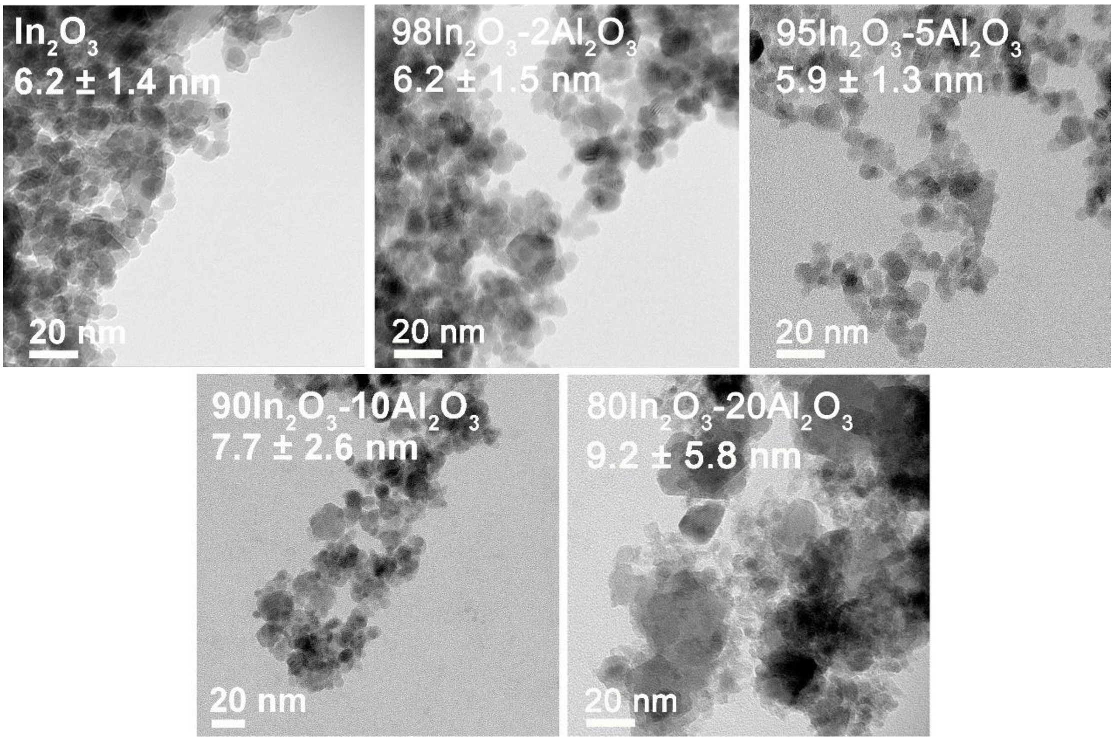

Figure S7. TEM images of the used In_2O_3-Al_2O_3 samples.

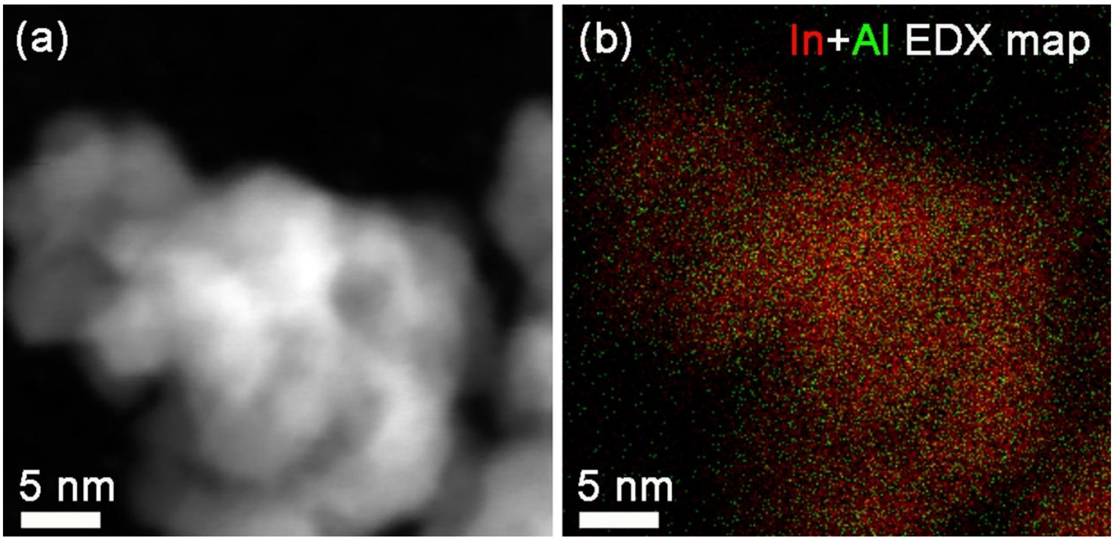

Figure S8. (a) HAADF image and (b) STEM-EDX elemental mapping of the used $95In_2O_3$-$5Al_2O_3$-IM sample (In red, Al green).

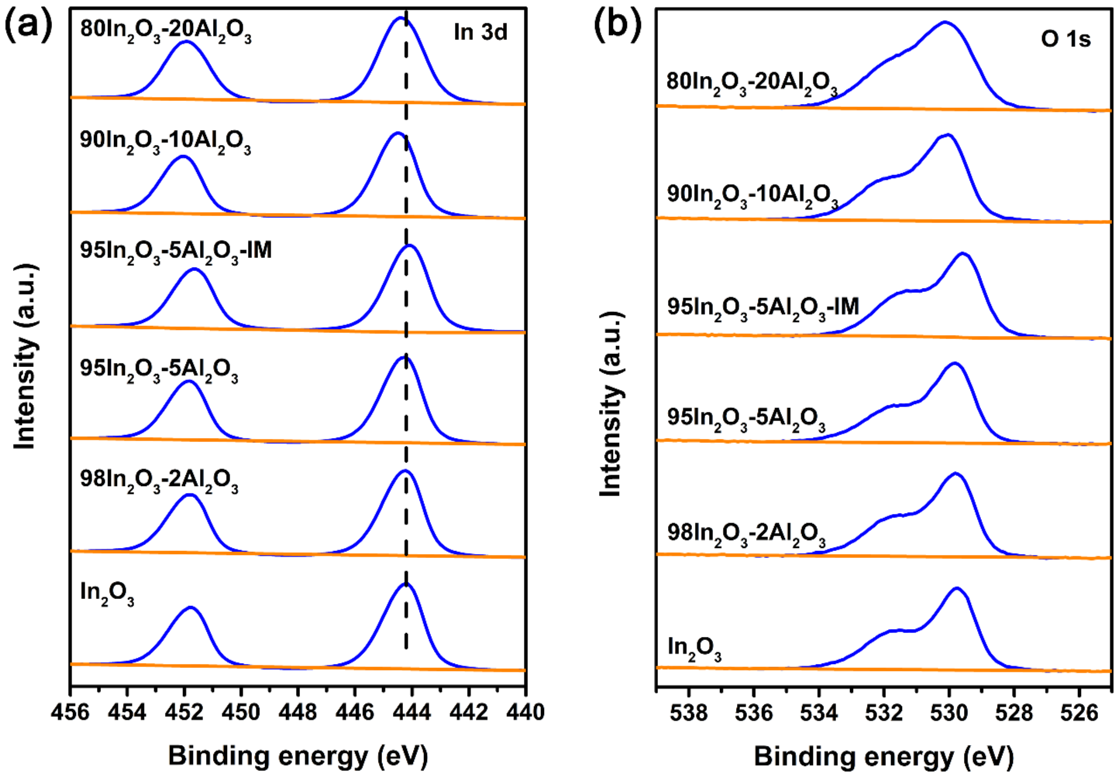

Figure S9. XPS spectra of (a) In 3d, (b) O 1s over the used In_2O_3-Al_2O_3 samples with different Al loading.

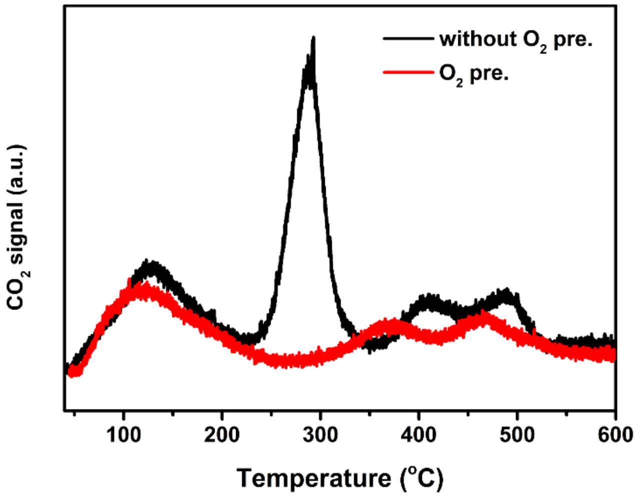

Figure S10. CO_2-TPD profiles of 95In_2O_3-5Al_2O_3 sample with/without O_2 pretreatment.

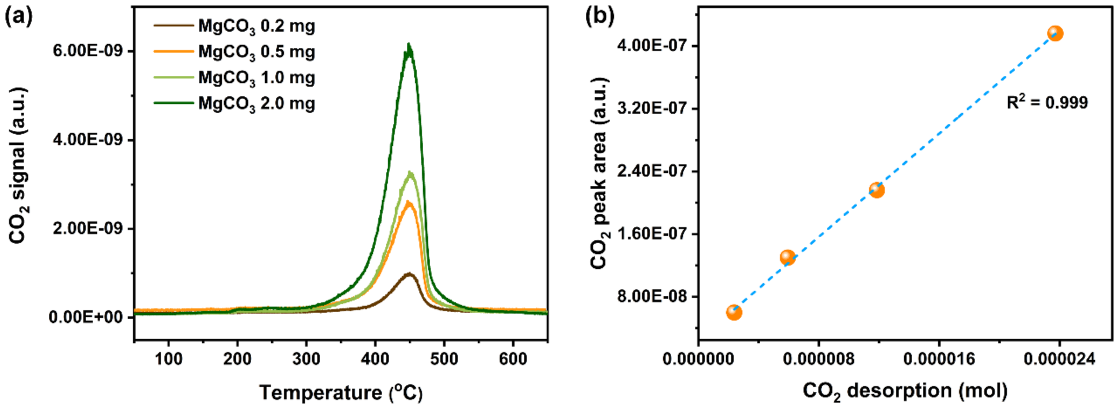

Figure S11. (a) TPD profiles of $MgCO_3$. (b) CO_2 peak area as a function of the amount of CO_2 formed during $MgCO_3$ decomposition.

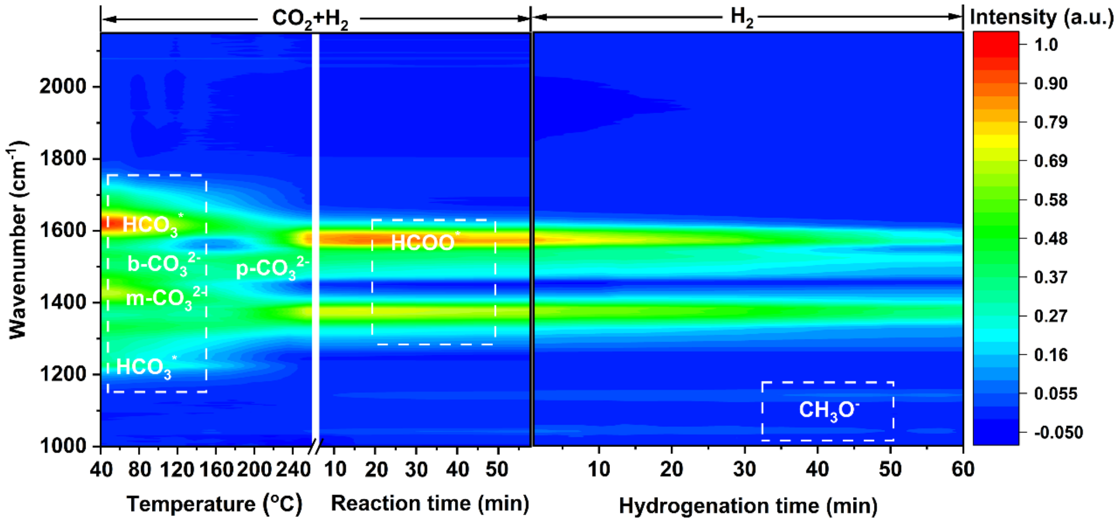

Figure S12. In situ FTIR as a function of temperature, reaction and hydrogenation time over In_2O_3. Reaction conditions: CO_2:H_2 =1:3, gas flow rate = 50 mL/min, *P* = 10 bar; Hydrogenation under pure H_2 (50 mL/min) at 260 °C and 1 bar.

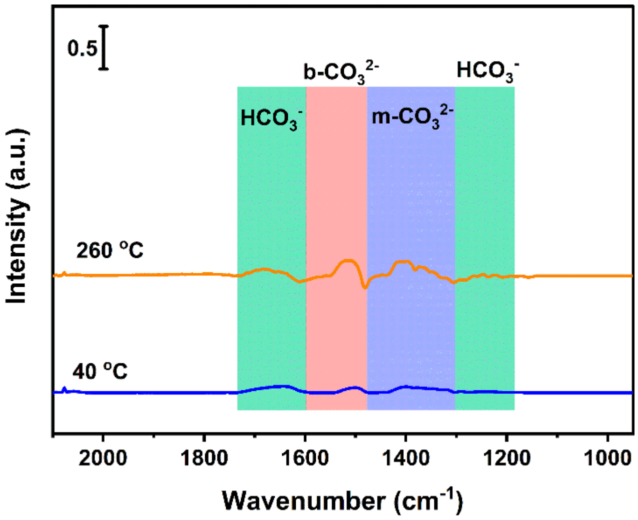

Figure S13. FTIR spectra at temperature of 40 and 260 °C over FSP-derived Al_2O_3. Reaction conditions: CO_2:H_2 =1:3, gas flow rate = 50 mL/min, P = 10 bar.

Table S1. H_2-TPR and CO_2-TPD data for In_2O_3-Al_2O_3 samples.

Sample	H_2 consumption (mmol · g_{cat}^{-1})[a]	CO_2 desorbed (μmol · g_{cat}^{-1})[b]	CO desorbed area (x 10^{-3})[c]
In_2O_3	0.35	16.1	0.7
$98In_2O_3$-$2Al_2O_3$	0.42	57.8	2.4
$95In_2O_3$-$5Al_2O_3$	0.44	118.8	3.5
$90In_2O_3$-$10Al_2O_3$	0.61	63.4	1.6
$80In_2O_3$-$20Al_2O_3$	0.64	69.5	1.8
$95In_2O_3$-$5Al_2O_3$-IM	0.90	66.4	2.5

[a] H_2 consumption is calculated by integrating the area of the reduction peak between 200 °C and 350 °C, and normalized by catalyst weight of the as-prepared In_2O_3-Al_2O_3 samples.
[b] The amount of CO_2 desorbed is calculated by integrating the area of the medium desorption peak between 200 and 350 °C based on CO_2-TPD results and calibrating with CO_2 produced in the decomposition of magnesium carbonate.
[c] Peak area integrated from 200 to 450 °C for CO desorption based on CO_2-TPD is calibrated by the decomposition of magnesium carbonate to exclude the CO fraction from CO_2 in MS.

Table S2. XPS spectra of O 1s data for as-prepared In_2O_3-Al_2O_3 samples

Sample	O(lattice)	O_v(%)[a]	OH (%)
In_2O_3	64	18	18
$98In_2O_3$-$2Al_2O_3$	64	22	14
$95In_2O_3$-$5Al_2O_3$	64	22	14
$90In_2O_3$-$10Al_2O_3$	69	14	17
$80In_2O_3$-$20Al_2O_3$	74	9	17
$95In_2O_3$-$5Al_2O_3$-IM	70	16	14

[a] The oxygen vacancy concentration of In_2O_3-Al_2O_3 catalysts can be calculated with C_{Ov} (%)= $A_{Ov}/(A_{O(lattice)}+ A_{Ov}+A_{OH})\times 100$ and the area of O_v can be calculated with

A_{Ov}= A_{Ov}- 1.5×RSF_O×A_{Al}/RSF_{Al} -3×RSF_O×$A_{Carbonate}$/RSF_C, where RSF_O, RSF_{Al} and RSF_C stand for the relative sensitivity factors of O, Al and C elements, respectively.

Table S3. Elemental composition of the as-prepared and used In_2O_3-Al_2O_3 samples.

Sample	Al (mol%)[a]	Al/(In+Al) (mol%)[b]	In/(In+Al) (mol%)[b]	Al/(In+Al) (mol%)[b]	In/(In+Al) (mol%)[b]
		As-prepared		Used[c]	
In_2O_3 (fresh)	0	0	100	0	100
98In_2O_3-2Al_2O_3	1.5	2.6	97.4	2.9	97.1
95In_2O_3-5Al_2O_3	4.6	4.0	96.0	7.4	92.6
90In_2O_3-10Al_2O_3	9.7	8.2	91.8	14.7	85.3
80In_2O_3-20Al_2O_3	19.8	18.3	81.7	34.4	65.6
95In_2O_3-5Al_2O_3-IM	4.7	3.9	96.1	3.3	96.7

[a] Determined by ICP-OES elemental analysis.

[b] Determined by XPS

[c] The samples are measured after CO_2 hydrogenation reaction (260 °C, 30 bar, $CO_2/H_2/N_2$ = 10/30/10 mL/min, 14 h).
